# Acquisition of Rab11 and Rab11-Fip2—A novel strategy for *Chlamydia pneumoniae* early survival

**DOI:** 10.1371/journal.ppat.1006556

**Published:** 2017-08-07

**Authors:** Katja Mölleken, Johannes H. Hegemann

**Affiliations:** Institute for Functional Microbial Genomics, University of Düsseldorf, Düsseldorf, Germany; McMaster University, CANADA

## Abstract

The initial steps in chlamydial infection involve adhesion and internalization into host cells and, most importantly, modification of the nascent inclusion to establish the intracellular niche. Here, we show that *Chlamydia pneumoniae* enters host cells via EGFR-dependent endocytosis into an early endosome with a phosphatidylinositol 3-phosphate (PI3P) membrane identity. Immediately after entry, the early chlamydial inclusion acquires early endosomal Rab GTPases including Rab4, Rab5, Rab7, as well as the two recycling-specific Rabs Rab11 and Rab14. While Rab5, Rab11 and Rab14 are retained in the vesicular membrane, Rab4 and Rab7 soon disappear. Loss of Rab7 enables the *C*. *pneumoniae* inclusion to escape delivery to, and degradation in lysosomes. Loss of Rab4 and retention of Rab11/ Rab14 designates the inclusion as a slowly recycling endosome—that is protected from degradation. Furthermore, we show that the Rab11/ Rab14 adaptor protein Rab11-Fip2 (Fip2) is recruited to the nascent inclusion upon internalization and retained in the membrane throughout infection. siRNA knockdown of Fip2 demonstrated that the protein is essential for internalization and infection, and expression of various deletion variants revealed that Fip2 regulates the intracellular positioning of the inclusion. Additionally, we show that binding to Rab11 and Fip2 recruits the unconventional actin motor protein myosin Vb to the early inclusion and that together they regulate the relocation of the nascent inclusion from the cell periphery to the perinuclear region, its final destination. Here, we characterize for the first time inclusion identity and inclusion-associated proteins to delineate how *C*. *pneumoniae* establishes the intracellular niche essential for its survival.

## Author summary

Here, we show for the first time how *Chlamydia pneumoniae* an obligate intracellular pathogen establishes its intracellular niche. After EGFR-dependent endocytosis into host cells, the nascent chlamydial inclusion acquires early endosomal membrane identity and the Rab GTPases Rab4, Rab5 and Rab7, as well as the recycling-specific Rab11 and Rab14. We show that Rab5, Rab11 and Rab14 are retained in the vesicular membrane, while Rab4 and Rab7 subsequently disappear. Thus, *C*. *pneumoniae* escapes lysosomal degradation by hiding in a recycling endosome vesicle. Furthermore, we show that the Rab11/Rab14 adaptor protein Rab11-Fip2 (Fip2), together with the unconventional actin motor protein myosin Vb, is recruited to the nascent inclusion. Both are essential for internalization and infection, as they regulate the intracellular positioning of the inclusion, which is essential for intracellular transport from the cell periphery to the perinuclear region. Here, we characterize for the first time inclusion identity and inclusion-associated proteins to understand how *C*. *pneumoniae* establishes the intracellular niche, which is essential for its survival.

## Introduction

*Chlamydia pneumoniae* is the causative agent of a variety of acute and chronic diseases of the upper and lower respiratory tract including pneumonia, asthma, bronchitis and sinusitis, and is associated with ~50% of cases of chronic obstructive pulmonary disease [1]. *C*. *pneumoniae*, like all other *Chlamydia* species, is an obligate intracellular pathogen whose infectious, metabolically inactive elementary bodies (EBs) adhere to host cells. The first contact occurs via an electrostatic glycosaminoglycan—OmcB interaction, followed by binding of the chlamydial adhesin and invasin Pmp21 to the epidermal growth factor receptor (EGFR) [2], [3]. Binding to EGFR results in receptor phosphorylation, which activates downstream signaling cascades and recruits the endocytosis adaptor proteins c-Cbl and Grb2 to the bacterial entry sites [3].

In a previous study we made the surprising observation that the internalized bacteria remain associated with activated EGFR even after reaching their final destination in the perinuclear region [3]. Typically, ligand-mediated activation of EGFR either leads to degradation of the receptor via the lysosomal pathway or its recycling to the plasma membrane [4]. The choice of pathway is regulated by the type and concentration of the ligand bound to the receptor [5]. Thus, in order to establish the early inclusion, *C*. *pneumoniae* must somehow intervene in EGFR-mediated events so as to avoid EGFR-triggered degradation or rerouting back to the plasma membrane.

The fate of every endocytic process is decided by the early endosome (EE) or sorting endosome (SE), which acts as a sorting station, in which assignment to the recycling or degradation pathway is orchestrated by the presence of various small Rab GTPases in endosome subdomains [6], [7], [8]. Besides their complement of specific Rab proteins, endosomal vesicles are defined by the phosphatidylinositide (PIP) composition of their membranes. The PIP composition is tightly regulated and coordinates localization of Rabs and PIP-binding Rab effector proteins to the endosomal membrane in order to regulate the endocytotic status of vesicles [9].

Maturation of a Rab5-positive EE into a Rab7-positive late endosome (LE) and the conversion of PI3P to PI(3,5)P in the LE membrane directs the late endosome to the lysosome. Rapid recycling of proteins to the plasma membrane directly from the EE is regulated by Rab4, while Rab11-positive recycling endosomes (RE) are first transported to the endosomal recycling compartment (ERC) localized near the microtubule-organizing center and the Golgi complex [10], [11], [12] before being recycled. Rab14, a member of the Rab11 subfamily, regulates maturation of early phagosomes and defines intermediate recycling compartments [13], [12]. Furthermore, progression along the endocytic route is dependent on Rab effector proteins and their interaction with motor proteins for transport along microtubules (dynein/kinesin) or actin (myosin) [14], [12]. Some Rabs share these effector proteins. Thus both Rab11 and Rab14 interact with Rab11-Fip2 (Fip2) and Rab11-Fip1A (RCP), which are class I Rab11 effectors [15], [16].

Many intracellular pathogens, especially obligate intracellular parasites like Chlamydia, face the same problem: they must modify the endosomal compartment they inhabit to guarantee successful infection. The most critical steps involve the remodeling of the primary vesicle, as this determines the future pathogen-specific niche within the cell. The whole life cycle, starting with the pathogen’s internalization into the host cell, is adapted to generate and maintain this single, pathogen-defined intracellular compartment. Pathogens like Salmonella or Legionella have evolved strategies to modify their specific vesicle identity by secreting effector proteins that either target Rab activity or effectors, or influence the PIP composition of the vesicular membrane, or use a combination of those strategies [17]. In the case of Chlamydia, several Rab proteins, e.g. Rab4, Rab10, Rab11 or Rab14, are known to interact with the mature chlamydial inclusion membrane from 2 h post infection (hpi) onwards. However, the chlamydia-specific vesicle-shaping events that occur during EB internalization, and determine subsequent inclusion formation and the fate of the compartment-modulating host proteins are not known [18], [19], [20], [21], [22], [23] [24].

In order to understand how, upon internalization via EGFR, *C*. *pneumoniae* manages to control its intracellular fate, we characterized the identity of the nascent inclusion from its formation until it reaches the nuclear region (0 min to 60 min p.i.). During this period, we determined the status of the inclusion—defined by EBs residing in an EGFR-positive vesicle—within the endosomal pathway by localization of endosomal marker proteins, characterization of the endosomal membrane’s phosphatidylinositide composition and identification of key interaction partners involved in internalization and intracellular transport. We found that *C*. *pneumoniae* resides in a PI3P-positive EE that acquires the Rab GTPases Rab5, Rab4, Rab7, Rab11 and Rab14. While Rab7 and Rab4 disappear over time, Rab11 and Rab14 are retained, together with their shared adaptor protein Fip2. This set of markers confers a recycling endosomal identity on the early inclusion. Fip2 itself, and its interactions with Rab11/Rab14 and with the unconventional myosin Vb motor protein, are essential for the internalization and intracellular positioning of the early inclusion. These data document for the first time how *Chlamydia pneumoniae* constructs its own intracellular niche by acquisition of specific Rabs and their interaction partners. This process is the key to survival of the bacteria within the host cell.

## Results

### Uptake of *C*. *pneumoniae* EBs is PI3K dependent

As Chlamydia internalization and inclusion maturation are poorly understood, we monitored phosphoinositide lipids in the membrane around the nascent inclusion from as early as 5 min post adhesion until it reaches the nuclear region at 60 min post infection (p.i.).

**Fig 1:**
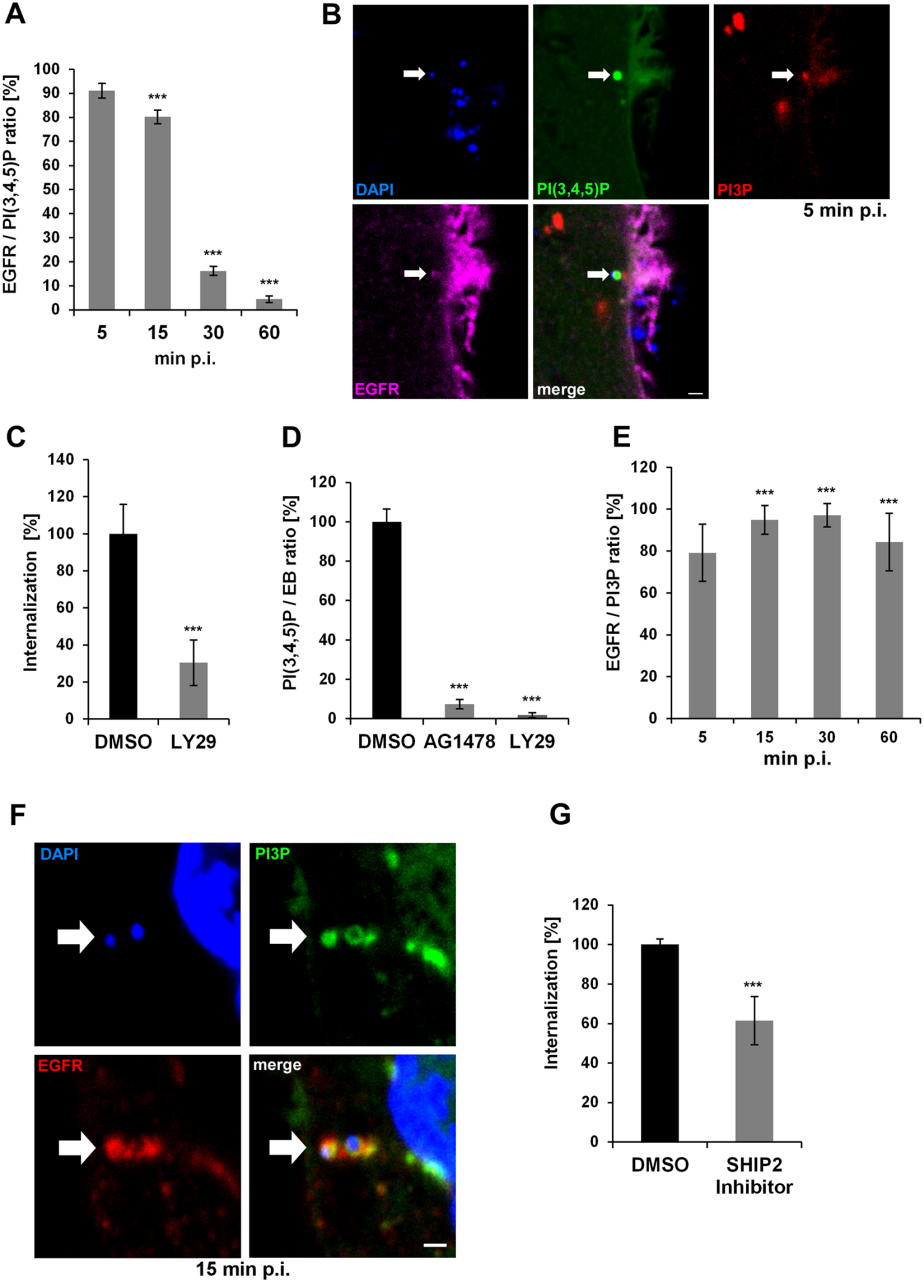
Entry of *C*. *pneumoniae* EBs is PI3K dependent. **(A)** Quantification of colocalization of PI(3,4,5)P and EGFR in cells transiently transfected with Btk-PH-GFP. Cells were infected at MOI 5, fixed with PFA at the indicated time points (5–60 min), and stained for endogenous EGFR using anti-EGFR, anti-rabbit Alexa594 and DAPI. Confocal images of 30 individual cells were used to analyze colocalization (n = 3). **(B)** The arrow marks a bacterial entry site at 5 min p.i. visualized by confocal imaging. The bacterial DNA stained with DAPI colocalizes with the “ring-like” signal of PI(3,4,5)P detected with Btk-PH-GFP. Furthermore, the entry site is marked by PI3P detected with mCherry-2xFYVE and the endogenous EGFR surrounding the internalized EB. **(C)** Quantification of *C*. *pneumoniae* internalization in cells pretreated with LY29 (50 μmol) for 2 h prior to infection. At 2 hpi internalization was measured by comparison numbers of internal and external EBs in 30 imaged cells (n = 4). **(D)** Quantification of EBs (stained with DAPI) colocalizing with PI(3,4,5)P (labeled by Btk-PH-GFP) at 5 min p.i. in cells pretreated with AG1478 (2 μmol) or LY29 (50 μmol) for 2 h (n = 3). **(E)** Quantification of colocalization of PI3P and EGFR in infected cells expressing GFP-2xFYVE. EGFR was stained as before (n = 3). **(F)** Colocalization of PI3P, EGFR and internalized EBs at 15 min p.i. by expression of GFP-2xFYVE and detection of EGFR. **(G)** Quantification of internalization in cells pretreated for 2 h with the SHIP2 inhibitor AS1949490 (10 μmol). *** *P* value ≤0.001. Bar 1 μm.

Ligand binding by EGFR leads to activation of the class 1 phosphoinositide 3-kinase (PI3K), which converts phosphatidylinositol 4,5-bisphosphate (PI(4,5)P), located in the inner leaflet of the plasma membrane, into phosphatidylinositol 3,4,5-triphosphate (PI(3,4,5)P). PI(3,4,5)P triggers the downstream Akt pathway, and is itself transformed into phosphatidylinositol 3-phosphate (PI3P) by the action of 5′-phosphatases such as SHIP1/2 and 4′-phosphatases like INPP4 at the plasma membrane [25]. Thus PI(3,4,5)P serves as the “starting lipid” for the regulation of endocytosis. When we monitored the *C*. *pneumoniae* infection process from 5 to 60 min p.i. in cells transfected with the PI(3,4,5)P-specific biomarker Btk-PH-GFP, we found that 91% of EBs that colocalized with EGFR at 5 min p.i. showed a ring of PI(3,4,5)P surrounding the bacterial DNA. Thus, this phospholipid indeed determines the point of entry (Fig 1A and 1B). Over the next 10 min, the number of signals decreased slightly to 80% (15 min p.i.) and had declined to 4% by 60 min pi, showing that although the infection is asynchronous the process of entry is completed within 1 h. When cells were preincubated with the PI3K inhibitor LY29 prior to infection, internalization of EBs was suppressed by 70% and infectivity decreased to 20% (Fig 1C, S1A Fig). The PI3 kinase is activated directly by the EB-mediated activation of EGFR, as revealed by quantifying the colocalization of PI(3,4,5)P with EBs at 5 min p.i. in cells pretreated with either LY29 or AG1478, a specific inhibitor of EGFR’s kinase activity (Fig 1D). Each inhibitor reduced the degree of colocalization of PI(3,4,5)P with EBs by more than 90%, indicating that upon binding and activation of EGFR the receptor activates PI3K, which results in the synthesis of PI(3,4,5)P at bacterial entry sites. This sequence of activation steps is essential for the uptake of *C*. *pneumoniae* into host cells.

To complete the process of endocytosis the PI(3,4,5)P generated upon activation of EGFR is dephosphorylated stepwise to PI3P by membrane-bound phosphatases. The appearance of this lipid indicates that a defined vesicle, the early endosome (EE), is formed. Indeed, in fixed cells we were able to detect PI3P colocalization with both EGFR and PI(3,4,5)P at the point of bacterial entry (Fig 1B). Underneath the PI(3,4,5)P signal at the plasma membrane we observed a diffuse PI3P signal indicating the PIP transition. Furthermore, we found PI3P-positive vesicles in close proximity to the bacterial entry site, implying that vesicle scission is followed by maturation and fusion with EEs. This was confirmed by live-cell imaging of cells expressing the PI(3,4,5)P marker Btk-PH-GFP and the mCherry-2xFYVE biosensor of PI3P. When these cells were infected with *C*. *pneumoniae* EBs labeled with Hoechst, we observed ring-like structures formed by PI(3,4,5)P colocalizing with bacterial DNA that recruit PI3P-positive vesicles (S1 Movie). By the time the bacteria are fully internalized the vesicular membrane has taken on a PI3P-positive identity, as revealed by live imaging (S2 and S3 Movies). Quantification of PI3P localization confirms that from 5 min p.i. onwards *C*. *pneumoniae* resides in an EGFR-positive EE (Fig 1E and 1F). These results are supported by the analysis of colocalization of the early endosomal Rab GTPase Rab5 or the early endosomal antigen 1 (EEA1) with EGFR-positive EBs. We observed similar patterns and numbers of colocalization, clearly marking chlamydia-containing vesicles as EEs, with Rab5 and EEA1 remaining associated with the inclusion membrane during the first 60 min p.i. (S1B and S1C Fig). These data indicate that bacterial contact first results in local PI(3,4,5)P production, which is converted to PI3P during bacterial uptake, and that the pinched-off chlamydial vesicle then fuses with additional small EEs to generate a fully mature EE.

To verify that *C*. *pneumoniae* relies on EGFR/PI3K-mediated progression into a PI3P/Rab5/EEA1-positive vesicle, we pretreated cells with a SHIP1/2-specific inhibitor, which blocks its phosphatase function and thus interferes with the generation of PI3P during endocytosis. SHIP inhibition resulted in a 40% reduction in numbers of internalized EBs and a 60% reduction in infectivity (Fig 1G, S1D Fig), indicating that PI3P formation and thus the EE is indeed essential for *C*. *pneumoniae* endocytosis and survival.

### Maturation of the early *C*. *pneumoniae* inclusion into an EE is dependent on Akt/PIKfyve activity

**Fig 2:**
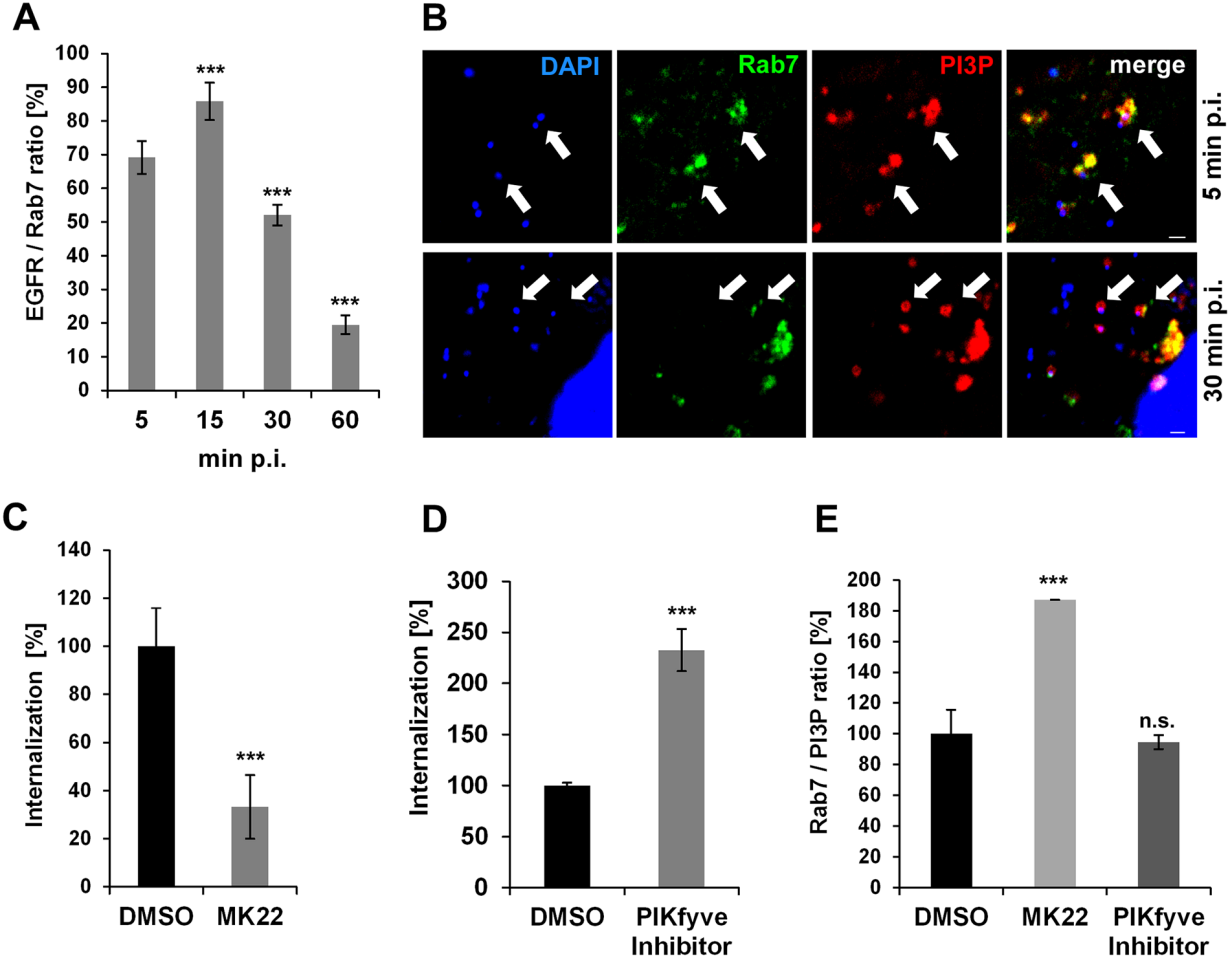
Development of early *C*. *pneumoniae* inclusion is dependent on Akt/PIKfyve activity. **(A)** Quantification of colocalization of Rab7 and EGFR with chlamydial EBs during the first hour p.i. in cells transiently transfected with GFP-Rab7 (n = 3). **(B)** Confocal images of GFP-Rab7 colocalizing with EBs in PI3P-positive endosomes (visualized by mCherry-2xFYVE and DAPI, respectively) at 15 min (top row) and 30 min p.i. (bottom row). White arrows indicate colocalization. Bar 1 μm. **(C–E)** Quantification of EB internalization in cells pretreated with the indicated inhibitor for 2 h prior to infection. Internalization was analyzed in 30 individual cells. **(C)** Entry of EBs into cells pretreated with the Akt inhibitor MK22 (3 μmol) or DMSO for 2 h prior to infection. Internalization was quantified microscopically at 2 h p.i. (n = 3). **(D)** Effects of pretreatment with the PIKfyve inhibitor YM201636 (800 nmol) for 2 h prior to infection on EB internalization (n = 3). **(E)** Quantification of GFP-Rab7 colocalization with EBs in PI3P-positive endosomes (visualized by mCherry-2xFYVE and DAPI) at 30 min p.i. in cells pretreated with MK22, the PIKfyve inhibitor or DMSO (n = 3). *** *P* value ≤0.001, n.s. *P* value <0.05.

Previous work by Coombes and Mahony revealed that activation of PI3K upon binding of *C*. *pneumoniae* is followed by activation of the Akt kinase [26]. During EGFR endocytosis Akt activates the PIKfyve kinase, which in turn directs EGFR-containing endosomes to lysosomes for degradation, via the classic pathway [27]. In contrast, inhibition of Akt activation leads to enhanced EGFR recycling. To further investigate the fate of the nascent inclusion, we looked at the distribution of the late endosomal marker Rab7, which is a key regulator in endo-lysosomal trafficking. Rab7 is recruited to sorting endosomes by a Rab5/Rab7 switch mechanism, and accumulation of Rab7 marks endosomes destined for the lysosome [28]. Intriguingly, we observed that, from the moment of entry (5 min p.i.) to the fully internalized *C*. *pneumoniae* EB at 15 min (defined by EBs within PI3P vesicles), colocalization of Rab7 with the EGFR-positive EBs increased from 70% to 85% (Fig 2A and 2B). Thereafter, however, the degree of colocalization decreased to 50% at 30 min p.i. and only 20% at 60 min p.i., at which time the inclusion is found near the nucleus. These findings indicate that, although Rab7 initially marks the inclusion, the degradation signal is progressively lost from EB-containing vesicles (Fig 2A and 2B).

Next, we analyzed the effects on the internalization and the identity of the *C*. *pneumoniae* endosome of Akt signaling and the activation of its downstream target PIKfyve kinase. The latter phosphorylates PI3P to PI(3,5)P, which is the PIP characteristic of the late endosomal membrane. When Akt signaling was inhibited by treating cells prior to infection with the Akt-specific inhibitor MK22, we observed a 70% reduction in internalization of EBs and a corresponding 65% reduction in infection (Fig 2C, S2A Fig). However, application of the inhibitor to cells expressing either GFP-Rab7 or GFP-Rab7 together with the PI3P sensor at 30 min p.i. was associated with a 2.7-fold increase in the number of EBs colocalizing with Rab7 compared to cells treated with solvent only (Fig 2E, S2C Fig). Thus, inhibition of the Akt pathway prior to infection results in less internalization and the internalized EBs are marked for degradation. Hence, triggering of Akt activity during early infection is essential for the establishment of the *C*. *pneumoniae* infection. Interestingly, when analogous experiments were performed with the inhibitor of PIKfyve activity, which blocks endosome maturation, resulting in enlarged EEs that cannot progress to late endosomes, we saw a massive increase in EB internalization (230%) and subsequent infection (Fig 2D, S2B Fig). However, in contrast to MK22, the PIKfyve inhibitor does not alter the level of vesicle-associated Rab7 seen in control cells (Fig 2E, S2C Fig). Together, these findings indicate that activation of Akt is required to enable *C*. *pneumoniae* to release Rab7 from EEs. Inhibition of the PIKfyve activity further enhances this effect by inhibition of endosome maturation.

### The early *C*. *pneumoniae* inclusion is a recycling endosome

**Fig 3:**
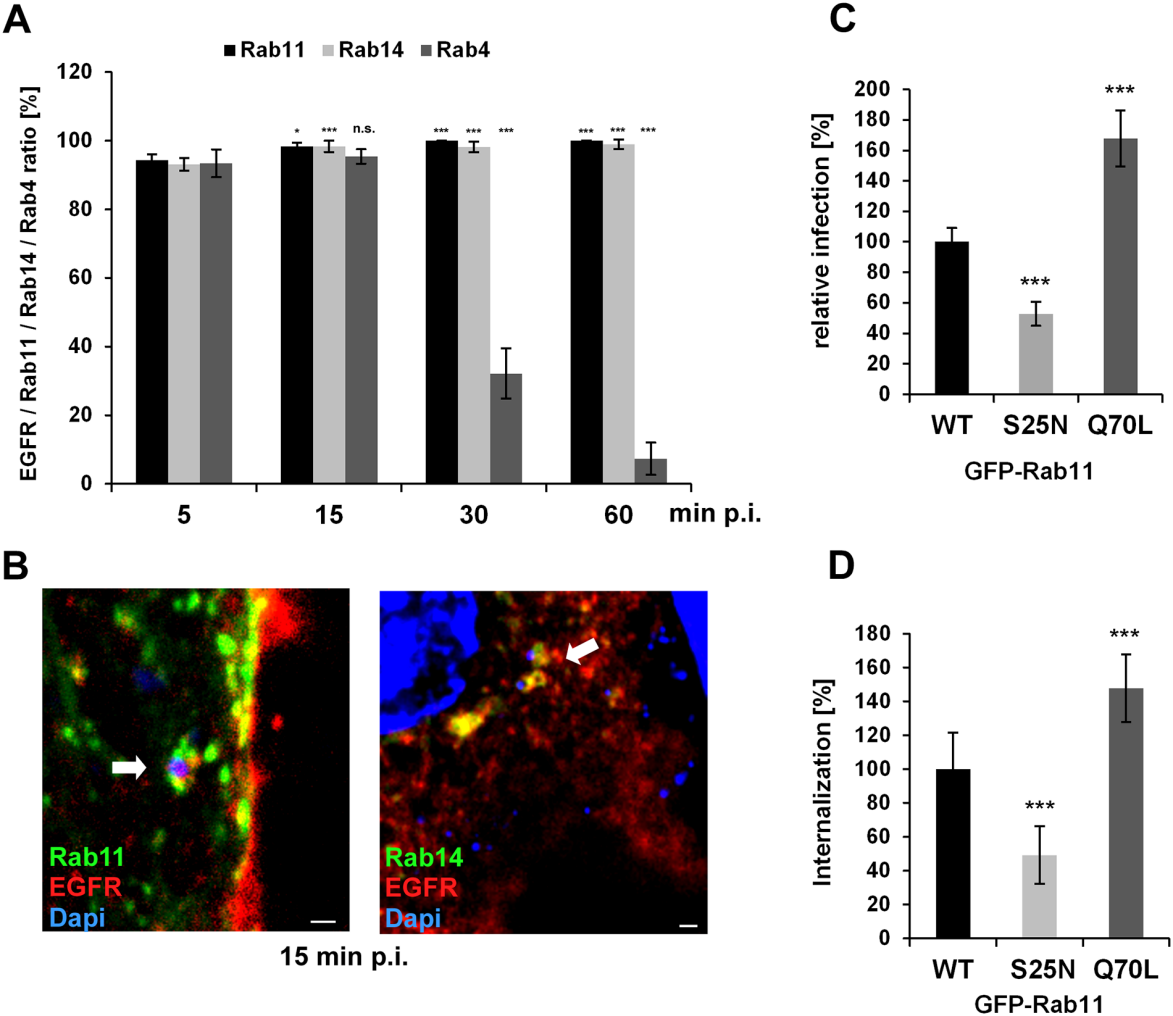
The early *C*. *pneumoniae* inclusion is a recycling endosome. **(A)** Quantification of colocalization of EGFR-positive *C*. *pneumoniae* EBs with GFP-Rab11, GFP-Rab14, GFP-Rab4 at 5–60 min p.i. in transiently transfected cells over the course of (n = 3). **(B)** Confocal images of colocalization of EBs (visualized with DAPI) with GFP-Rab11 and EGFR (left), or with GFP-Rab14 and EGFR (right) at 15 min p.i. White arrows indicate colocalization. Bar 1μm. **(C, D)** HEp-2 cells stably expressing wild-type GFP-Rab11 or the dominant-negative (S25N) or constitutively active (Q70L) GFP-Rab11 variant. **(C)** Quantification of EB internalization at 2 hpi in 30 individual cells (n = 4). **(D)** Quantification of infection at 48 hpi in 40 visual fields (n = 4). *P* values: * ≤ 0.1; *** ≤0.001. n.s.–not significantly different.

As the early endosome stands at the crossroad between degradation and recycling, we analyzed the nature of the *C*. *pneumoniae* inclusion in more detail. Thus far, we have seen that Rab7 is lost from the inclusion and degradation is avoided. We therefore focused on various marker proteins for the endosomal recycling pathway, as this is most likely to be the path taken. Again, we looked at the process from adhesion to the final deposition of EBs in EGFR-positive vesicles near the nucleus in cells expressing GFP-tagged Rab4, Rab11 and Rab14 (Fig 3A and 3B). While Rab4 promotes fast recycling directly at the plasma membrane, Rab11 is the key regulator of slow recycling via transport of endosomal vesicles to the perinuclear ERC [11], [12]. Rab14 is a more specialized member of the Rab11 subfamily, which defines intermediate recycling compartments [13], [12].

Interestingly, already at 5 min p.i. about 95% of all EGFR-positive EBs colocalized with all three Rab proteins (Fig 3A and 3B, S3 Fig). This high colocalization frequency remained stable until 15 min p.i., when internalization is complete. But, as in the case of Rab7, significant loss of Rab4 from the inclusion is apparent by 30 min p.i. (39% colocalization), and at 60 min only approx. 5% Rab4 colocalization is observed (Fig 3A, S3 Fig). In contrast, Rab11 and Rab14 remain stably associated with EGFR, and at 60 min p.i. When we co-expressed Rab11 together with Rab4 or Rab14, the nascent inclusion (15 min p.i.) was positive for all Rab proteins, but from 30 min onwards, Rab4 disappeared (S3B Fig). These data indicate (i) that the recycling pathway is the route *C*. *pneumoniae* takes after internalization, and (ii) the bacteria preferentially exploit intracellular transport pathways regulated by Rab11/Rab14 and prevent fast recycling back to the plasma membrane by release of Rab4.

As Rab11 is acquired by the nascent *C*. *pneumoniae* inclusion from the moment of entry and is the classical Rab GTPase that defines the recycling route in the host cell, we analyzed whether the activity of Rab11 is important for infection and internalization. We therefore generated HEp-2 cells stably expressing GFP fusions of wild-type Rab11, the constitutively active Rab11Q70L or the dominant-negative Rab11S25N [29]. In comparison to overexpression of WT-Rab11, expression of Rab11S25N reduced internalization of bacteria by 49% and infection by 52% (Fig 3C and 3D). In contrast, high expression of Rab11Q70L boosted both internalization and infection by 147% and 167% respectively. Taken together, these observations imply that active Rab11 is required for the early processes leading to a successful infection.

### The early *C*. *pneumoniae* inclusion recruits the Rab11/Rab14 adaptor Fip2

The recycling system of the cell is a complex network with vesicles shuttling between the cell periphery and the endocytic recycling compartment (ERC) in the perinuclear region [10], [30], [31]. All transport pathways rely on the association of Rabs with specific Rab-interacting proteins. One of these proteins is Rab11-Fip2 (Fip2), a Rab11 effector that binds preferentially to PI(3,4,5)P, which regulates the transport of vesicles from the plasma membrane to the ERC or vice versa [16], [32], [33]. Fip2 is known to be important for the internalization and recycling of EGFR [34]. Interestingly, Fip2 is also bound by Rab14 [35].

**Fig 4:**
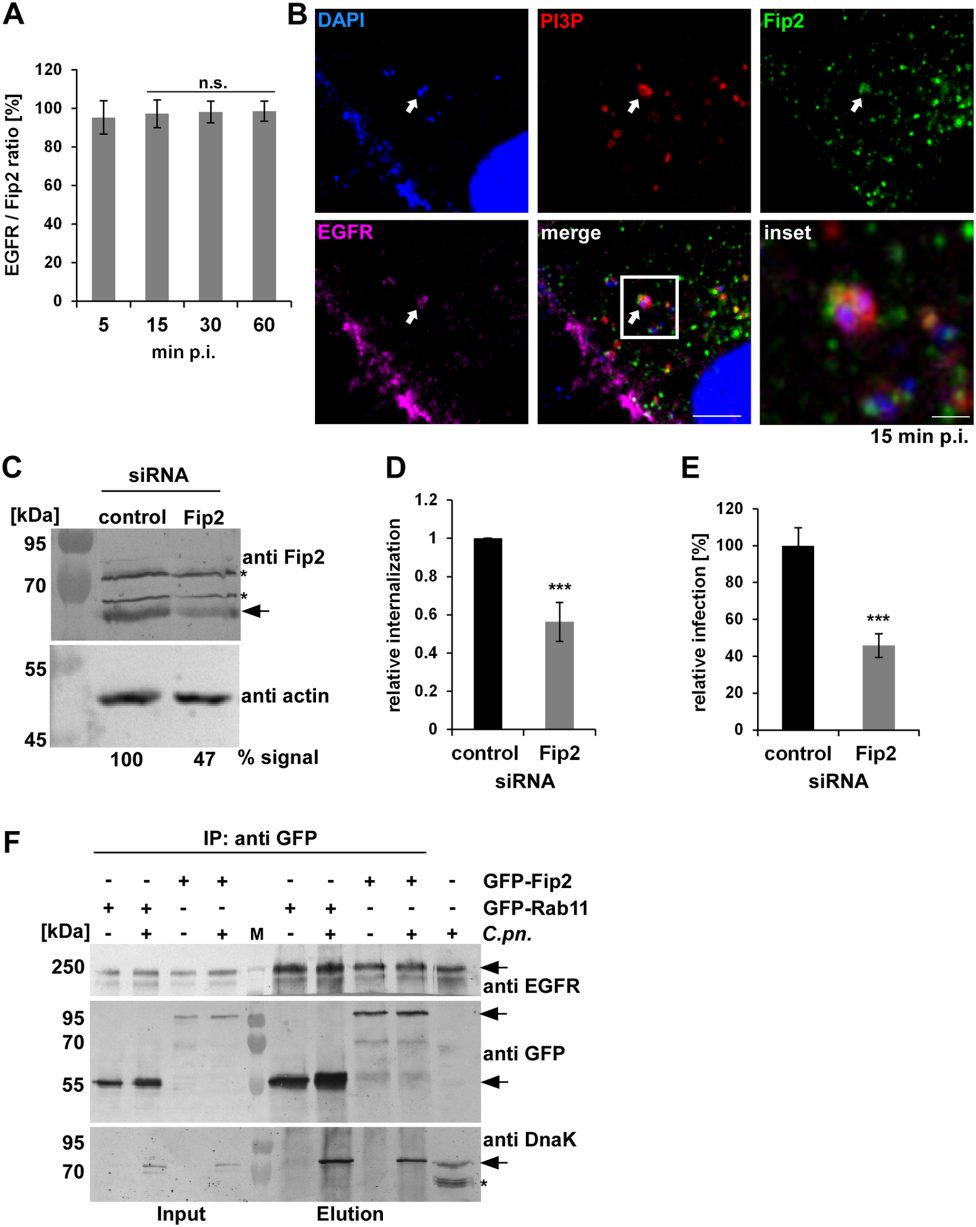
The Rab11/Rab14 adaptor Fip2 is recruited to early *C*. *pneumoniae* inclusions. **(A)** Quantification of colocalization of Fip2 and EGFR with chlamydial EBs at 5–60 min p.i. in cells transiently transfected with GFP-Fip2 (n = 3). **(B)** Confocal images of colocalization of EBs (stained by DAPI) with GFP-Fip2, PI3P and EGFR (visualized by mCherry-FYVE and anti EGFR and anti-rabbit Alexa647, respectively) at 15 min p.i. White arrows indicate colocalization, the white box marks the area enlarged in the inset. Bar 10 μm. Bar in inset 1 μm. **(C)** Immunoblot analysis of cells transiently transfected for 72 h with control or Fip2 siRNA. Samples were lysed in phospho-Lysis buffer, subjected to SDS/PAGE and probed with antibodies against Fip2 to monitor knockdown; β-actin served as the loading control. The pixel intensity of bands was analyzed with ImageJ. The arrow marks the Fip2 protein band, unspecific bands detected by the Fip2 antibody are indicated by asterisks. **(D, E)** Quantification of EB internalization or infection after transfection for 72 h with control or Fip2 siRNA. **(D)** Relative internalization levels were measured by q-PCR of isolated DNA using primers specific for human GAPDH and chlamydial 16S rRNA at 2 hpi (n = 6). **(E)** Quantification of infection analyzed microscopically as described before (n = 4). **(F)** Co-IP of HEp-2 cells stably expressing GFP-Rab11 or GFP-Fip2 after infection for 15 min. Chlamydia-containing endosomes were isolated and subjected to immunoprecipitation using a GFP antibody coupled to ProteinG Dynabeads. Input and elution samples from IP and a control sample of cells infected with *C*. *pneumoniae* for 72h (last lane) were fractionated by SDS/PAGE and probed with anti-EGFR, anti-GFP and anti-DnaK antibodies. Arrows mark specific protein bands, the asterisk indicates unspecific bands detected in the infected cells by the DnaK antibody. *** *P* value ≤0.001.

When we looked at the distribution of Rab11-Fip2 during the early phase of infection, we detected 95% colocalization of Rab11-Fip2 with EGFR/PI3P-positive endosomes containing chlamydial EBs at 5 min p.i., and this was confirmed by live-cell imaging of cells expressing GFP-Fip2 and either EGFR-mCherry or mCherry-2xFYVE (Fig 4A and 4B, S4, S5 and S6 Movies). This association was stably maintained up to 60 min p.i. Fip2 was not only present on the early inclusion, but was found to be associated with the *C*. *pneumoniae* inclusion membrane throughout the infection cycle and colocalized there with Rab11 and Rab14 (S4A and S4D Fig). This is in clear contrast to the *C*. *trachomatis* L2 infection, where Fip2 colocalizes with the inclusion from 2 h p.i. onwards but has disappeared by 24 h p.i. [36]. Coexpression studies of Fip2 with Rab11 or Rab14 revealed the same colocalization pattern, indicating that acquisition of the Rab11/Rab14 adaptor protein is important for the establishment of a *C*. *pneumoniae* infection (S4A Fig). Indeed when we suppressed Fip2 synthesis by siRNA we saw a 44% reduction in internalization of EBs and a 65% drop in infection (Fig 4C, 4D and 4E). However, the inclusions formed looked normal in shape and size. Again, this differs from the *C*. *trachomatis* infection, where depletion of Fip2 results in smaller inclusions [36].

Thus, Fip2 is essential for internalization and infection, and is found on the nascent inclusion, where it colocalizes with Rab11/Rab14. To further bolster this finding, we infected cells that stably expressed either GFP-Rab11 or GFP-Fip2 with *C*. *pneumoniae* EBs for 15 min. The cells were then lysed in such way that Chlamydia-containing vesicles remained intact. When we subsequently performed immunoprecipitation of GFP-Rab11 or GFP-Fip2 we were able to co-isolate endogenous EGFR and EBs (Fig 4F). We confirmed these results by using cells transiently co-transfected with EGFR-Myc and GFP-Fip2 or GFP-Rab11 and performing the same experimental procedure (S4B and S4C Fig). When we isolated EGFR-Myc using an anti-Myc antibody, we again detected Fip2 and Rab11, as well as chlamydial DnaK, in these samples. Hence, early chlamydial endosomes enriched at 15 min p.i. indeed contain EGFR, Rab11 and Fip2.

### The Rab11 binding domain of Fip2 is essential for the *C*. *pneumoniae* infection

**Fig 5:**
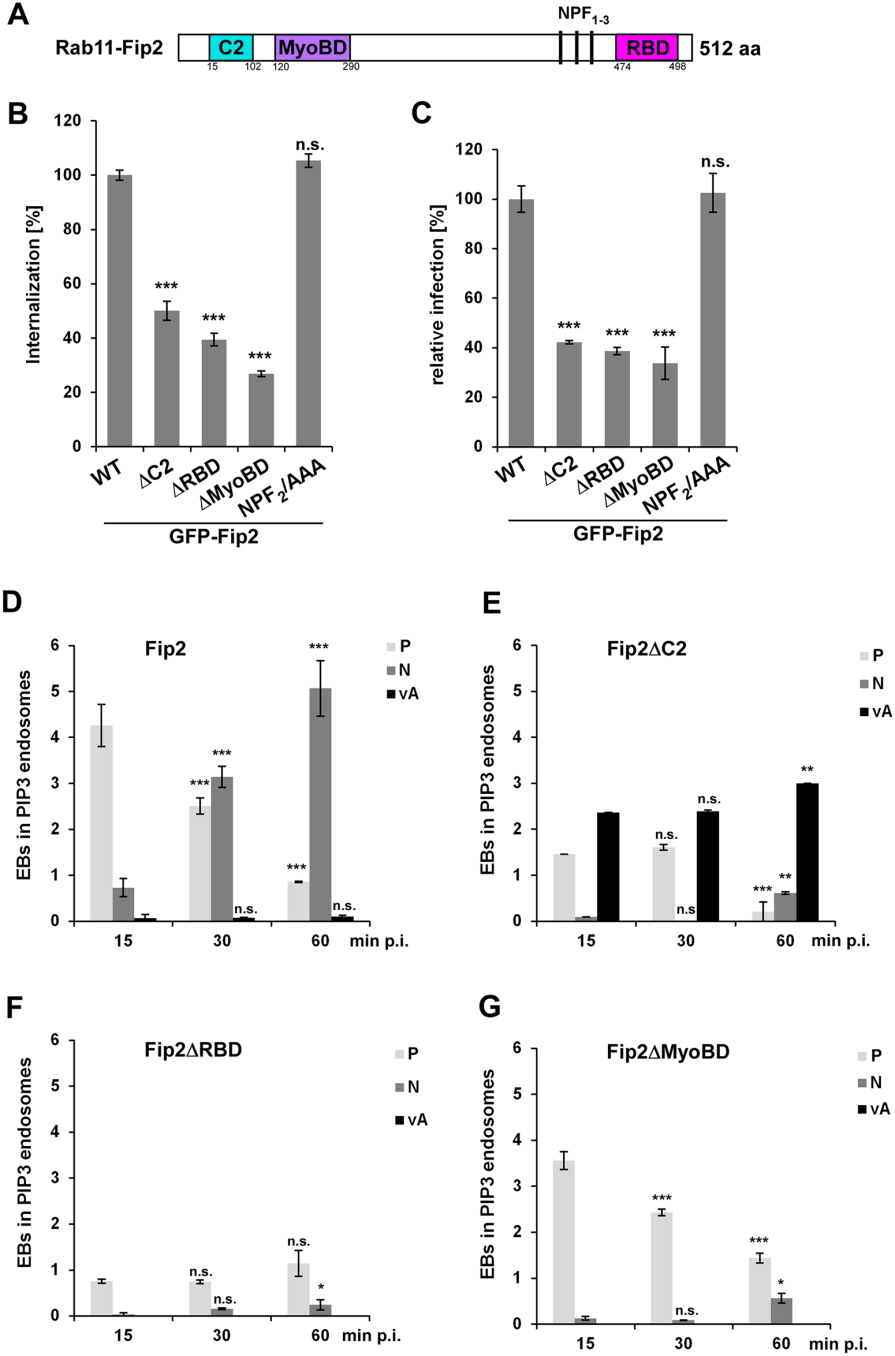
The Rab11-binding domain of Fip2 is essential for *C*. *pneumoniae* infection. **(A)** Schematic representation of Fip2. **(B, C)** Quantification of EB internalization **(B)** or chlamydial infection **(C)** in HEp-2 cells stably expressing the indicated GFP-Fip2 mutant variants. **(B)** Analysis of *C*. *pneumoniae* EB internalization at 2 hpi in 30 individual cells (n = 3). **(C)** Quantification of infection based on the numbers of inclusions found in 40 visual fields at 48 hpi (n = 4). **(D-G)** Colocalization analysis of EBs in PI3P-positive endosomes (visualized with mCherry-2xFYVE and DAPI) in HEp-2 cells stably expressing GFP-Fip2 mutant variants at 15 min to 60 min p.i. For each time point the intracellular distribution of inclusions was assessed in 30 cells and the localization pattern was classified as follows: P, endosome in the cell periphery; N, endosomes in the perinuclear region; vA, vesicular aggregates in cell periphery. **(D)** Wild-type GFP-Fip2. **(E)** GFP-Fip2ΔC2. **(F)** GFP-Fip2ΔRBD. **(F)** GFP-Fip2ΔMyoBD. *** *P* value ≤0.001, ** *P* value ≤0.01, * *P* value ≤0.05, n.s. *P* value ≤0.01.

Having shown that Fip2 is recruited to the nascent inclusion at 5 min p.i. and is essential for internalization and the subsequent infection, we generated stable cell lines overexpressing different GFP-Fip2 mutant variants and analyzed their effects on the infection. The mutations assessed involved deletions of functional domains such as the C2 domain essential for lipid binding, the RBD domain for binding to Rab11 and the MyoBD domain for binding myosin (Fig 5A). We also included a point mutation in one of the asparagine-proline-phenylalanine (NPF) motif needed for interaction of Fip2 with proteins containing an EHD domain [37], [38]. EHD proteins are thought to be involved in regulating endocytic transport [39].

All mutants except the NPF-to-AAA variant showed reduced internalization compared to WT-Fip2-expressing cells, with 50% reduction for Fip2ΔC2, 61% for Fip2ΔRBD and 74% for Fip2ΔMyoBD (Fig 5B). The reductions in internalization in Fip2ΔC2-, Fip2ΔRBD- and Fip2ΔMyoBD-expressing cells correlated with the rate of infection, which again was unchanged for NPF_2_/AAA relative to wild-type Fip2 (Fig 5C).

For further analysis of the impact of Fip2 deletion variants on infection, the subcellular localization of the EB-containing early endosomes (PI3P-positive) from 15 to 60 min p.i. was determined. We distinguished three major localization patterns and defined them as PI3P-positive endosomal vesicles in the cell periphery (P), endosomal vesicles at the nucleus (N) and vesicular aggregates in the cell periphery (vA) (Fig 5D–5G). In WT-Fip2-expressing cells, the majority of Chlamydia-containing vesicles were found in the cell periphery during the first 15 min, while at 30 min p.i. equal numbers of vesicles were found in the periphery and next to the nucleus (Fig 5D). At 60 min the majority of the EB endosomes had reached the nucleus (Fig 5D). Interestingly, however this pattern is not reproduced in the other Fip2 variants tested. Overexpression of Fip2ΔC2, a variant that is unable to bind membrane lipids, resulted in fewer internalized EBs at 15 min p.i.; however, those internalized were distributed into normal vesicles and vesicular aggregates that localized to the cell periphery (Fig 5E). At 30 min p.i. this distribution remained stable and again no EBs were found at the nucleus, while at 60 min p.i. the majority of EBs was found in highly clustered vesicular aggregates in the cell periphery and only a few reached the nucleus (Fig 5E). Thus, Fip2ΔC2 is largely unable to facilitate transport of the EBs towards the nucleus, and the bacteria are instead retained in vesicular aggregates of endosomal character.

In cells expressing Fip2ΔRBD, which lacks the Rab11-binding domain, the protein was distributed all over the cytoplasm and the internalization of bacteria was reduced by approx. 80% (Fig 5F). Over the course of 60 min the distribution of bacteria-positive endosomes again remained restricted to the cell periphery and almost no EBs reached the nucleus. Thus, this variant is clearly defective in internalization and intracellular transport. Overexpression of Fip2ΔMyoBD, which carries a deletion in the domain required for binding to the motor protein myosin Vb, also resulted in retention of bacterial endosomes in the cell periphery (Fig 5G). Only a few EBs reached the peri-nucleus at 60 min p.i. These results indicate that the interaction of Fip2 with Rab11 and myosin Vb is essential for entry into host cells and the subsequent transport towards the perinuclear region, while deletion of the C2 domain causes EBs to be retained in vesicular aggregates.

Thus far, we have shown that internalization and intracellular transport of EBs towards the nucleus is impaired in all Fip2 deletion variants (Fig 5D–5G). This also affects the development and positioning of the late inclusion as, depending on the expressed Fip2 variant, inclusions are smaller and localize further from the nucleus than in control cells (S5A–S5C Fig). Taken together, these results suggest a global function for Fip2 during early and late stages of infection.

### Acquisition of myosin Vb by the Rab11-Fip2 adaptor protein is essential for the *C*. *pneumoniae* infection

The findings detailed above demonstrate that internalization of *C*. *pneumoniae* and the subcellular localization of both the early and the late inclusion is dependent on the function of Rab11-Fip2, and that early and late inclusions colocalize with Rab11 and Rab14. Movement of Rab11-positive vesicles is achieved by interaction of Fip2 with motor proteins such as myosin Vb. This unconventional myosin is an actin-binding motor protein, which tethers endosomal vesicles to the cortical actin cytoskeleton [40], [41]. The correct distribution of RE vesicles is important for the organization and function of the recycling endosome, as overexpression of a dominant-negative variant of myosin Vb, MyoVbtail, impairs recycling of REs by causing vesicles to accumulate in the perinuclear region. These retain Rab11 and Fip2, which suggests that peripheral vesicles are released from cortical actin and accumulate near the nucleus in the absence of the myosin Vb function [41].

**Fig 6:**
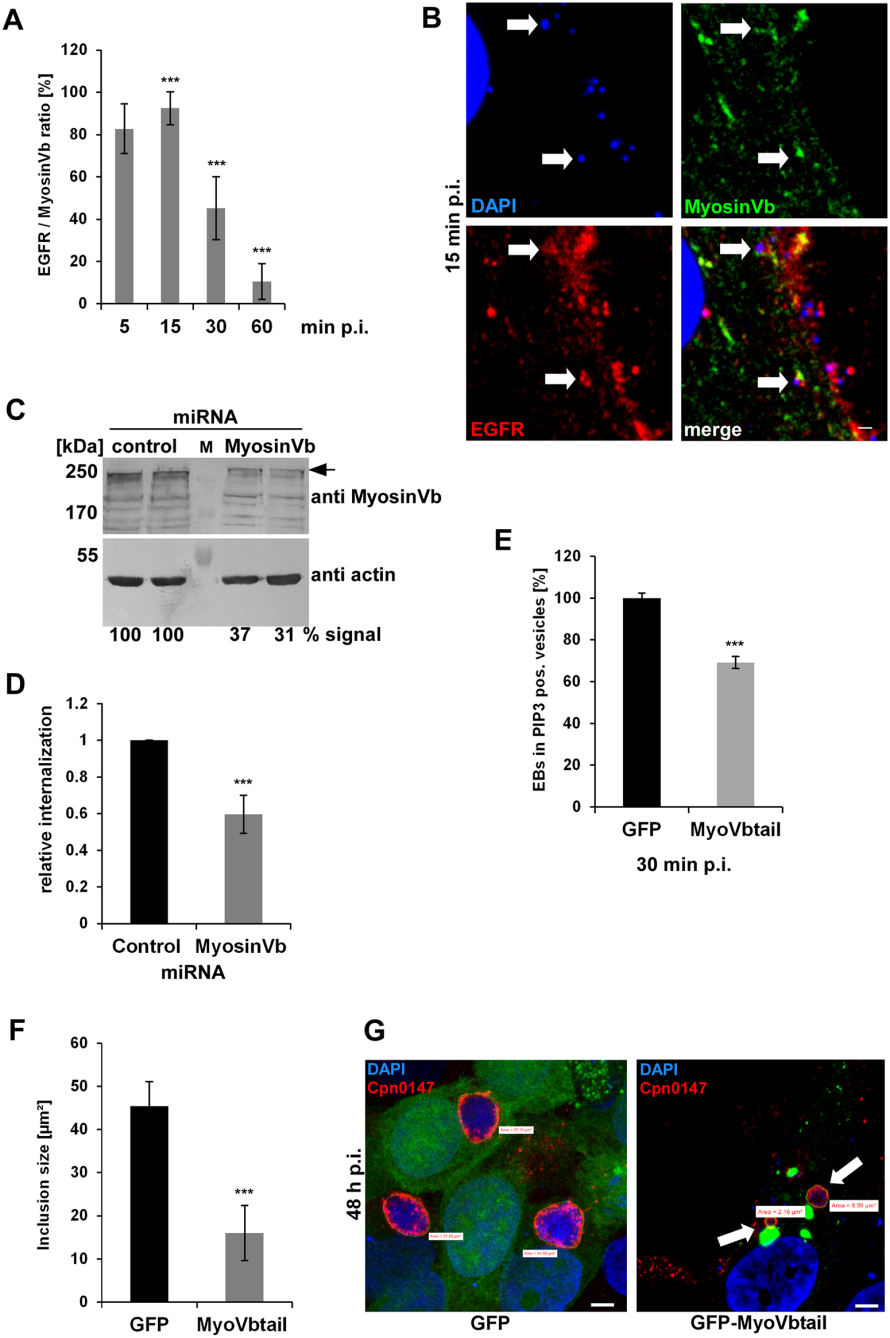
The acquisition of myosin Vb by the Rab11-Fip2 adaptor protein is essential for the *C*. *pneumoniae* infection. **(A)** Quantification of colocalization of EGFR-positive EBs with GFP-MyosinVb from 5 min to 60 min p.i. (n = 3). **(B)** Confocal images of colocalization of GFP-MyosinVb and EGFR with *C*. *pneumoniae* EBs at 15 min p.i. White arrows indicate colocalization. Bar 1 μm. **(C)** Immunoblot analysis of cells transiently transfected for 72 h with control or myosin Vb miRNA plasmids and lysed in phospho-Lysis buffer. Samples were fractionated by SDS/PAGE and probed with antibodies against myosin Vb to monitor knockdown; β-actin served as loading control. The pixel intensity of bands was analyzed with ImageJ. The arrow marks the specific myosin Vb band. **(D)** Relative internalization of EBs into cells transfected for 72 h with control or MyosinVb miRNA plasmids was measured by q-PCR at 2 hpi as described in the legend to Fig 4 (n = 6). **(E)** Quantification of internalization of EBs into PI3P endosomes at 30 min p.i. Confocal images of 30 individual cells transiently expressing mCherry-2xFYVE and GFP or GFP-MyoVbtail were analyzed (n = 3). **(F)** Quantification of the inclusion diameter in cells expressing in GFP or GFP-MyoVbtail cells at 48 hpi. Inclusions were stained with anti-Cpn0147 and anti-rabbit Alexa594 and their diameters were measured in 30 individual cells (n = 3). *** *P* value ≤0.001, n.s. *P* value ≤0.01 **(G)** Confocal images of cells quantified in (F). Arrows mark the smaller inclusions in cells expressing GFP-MyoVbtail. Green lines mark the outline of the inclusions used for quantification. Bar 5 μm.

When we analyzed colocalization of myosin Vb with EGFR-positive EBs in cells transiently transfected with GFP-MyosinVb, very few cells expressed the protein. In such cells 85% and 91% of Chlamydia-containing endosomes colocalized with myosin Vb at 5 min and 15 min p.i., respectively. Colocalization then decreased over time to 45% at 30 min and 10% at 60 min p.i., indicating that the motor protein is specifically associated with internalizing EBs (Fig 6A and 6B). Next, we generated a knockdown of the endogenous myosin Vb protein by miRNA expression (achieving between 63% and 69% reduction in protein level) and observed a 40% reduction in numbers of internalized EBs (Fig 6C and 6D). These results clearly show that the motor protein is essential for internalization of *C*. *pneumoniae*.

To further substantiate our findings we infected cells expressing the dominant-negative GFP-MyoVbtail, which showed the previously described aggregation of endosomes that retained both Rab11 and Fip2 close to the nucleus [42], [43]. GFP-MyoVbtail-expressing cells infected with *C*. *pneumoniae* for 30 min showed a 34% reduction in EBs endocytosed into PI3P-positive endosomes compared to the GFP control (Fig 6E). However, the endosome-harboring bacteria colocalized with the truncated myosin protein (S7 Movie) indicating that the latter is still recruited to the internalized Chlamydia and is important for the entry process. When we then analyzed the infection at 48h p.i., the GFP-MyoVbtail-expressing cells showed contained fewer inclusions, and these were 65% smaller in average diameter than those in GFP control cells (Fig 6F and 6G). Taken together, our data show that *C*. *pneumoniae* internalization and intracellular growth is dependent on the function of the actin-based motor protein myosin Vb.

## Discussion

As an obligate intracellular pathogen like all Chlamydia, *C*. *pneumoniae* must gain entry to a host cell to complete its intracellular life cycle and produce infectious progeny. Internalization, the establishment of the early inclusion and its intracellular transport to the perinuclear region are therefore the first essential steps for successful infection. These molecular processes occur within the first 60 min after host cell entry, and are poorly understood. The aim of this study was to gain detailed insight into the course of events during this period, which we define as ‘early infection’ (from 5 min to 60 min p.i.).

**Fig 7:**
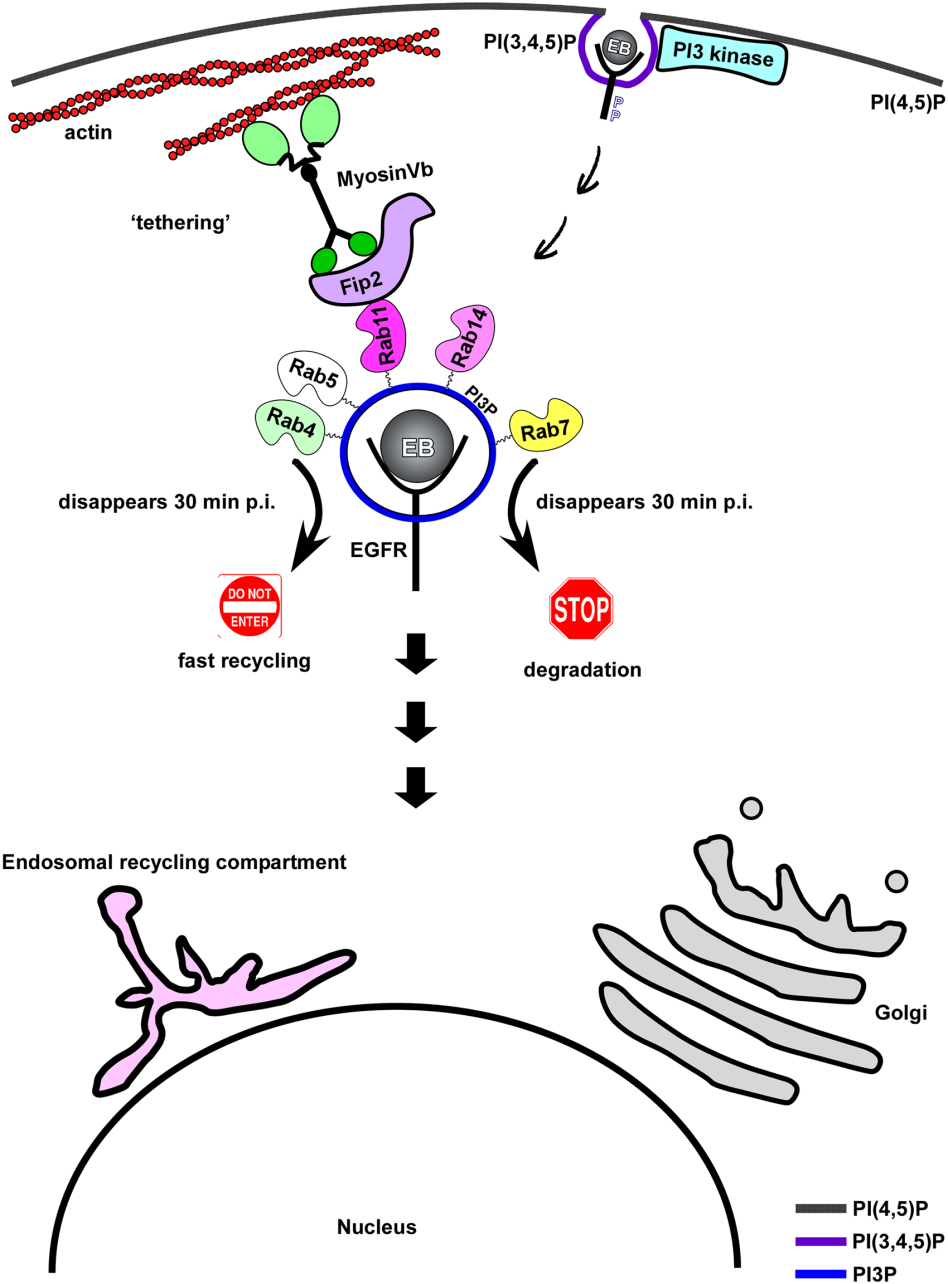
Model of early internalization and establishment of intracellular niche. The model shows that binding and internalization of *C*. *pneumoniae* EBs is mediated by EGFR and requires receptor activation. Activated EGFR stimulates the PI3 kinase located at the plasma membrane, thus generating PI(3,4,5)P, which is converted to PI3P. The internalized EB remains in a PI3P-positive endosome, which acquires several Rab GTPases e.g. Rab4, Rab5, Rab7, Rab11 and Rab14. Loss of Rab4 and Rab7 subsequently mark the early inclusion as a recycling endosome. In addition the Rab11/Rab14 adaptor protein Rab11-Fip2 is recruited, which is essential for intracellular positioning of the inclusion. This is achieved by interaction of Fip2 with the actin-based motor protein myosin Vb.

Our previous work had shown that *C*. *pneumoniae* binds to, and activates EGFR, which then mediates endocytosis of the bacteria into the cell [3]. The evidence presented here demonstrates that the resulting *C*. *pneumoniae*-containing vesicle develops into an EE (S2 and S3 Movies). The question is how Chlamydia controls this EGFR-dependent process, as the endocytosed receptor can subsequently be either degraded or recycled (Fig 7). Our data indicate that Chlamydia blocks maturation of EEs into LEs, thereby avoiding delivery to the lysosome and degradation. Instead, by remaining in PI3P-positive endosomes, which subsequently acquire the GTPases Rab11/Rab14 accompanied by their adaptor proteins, it becomes sequestered in recycling endosomes. Thus, *C*. *pneumoniae* establishes infection by disguising its inclusion as a recycling endosome.

Dissection of the different phases of internalization revealed that binding of EBs to cells leads to the activation of host PI3 kinase, which results in enrichment for PI(3,4,5)P in the host cell membrane at bacterial entry sites, which is dependent on EGFR activity (Fig 1, S1 Fig). Activation of PI3 kinase is essential for entry into the cell and for the specification of the Chlamydia-containing endosome as an early endosome (Figs 1 and 7, S1 Fig). Thus far, the PI3 kinase activation had only been shown to be important during early and late *C*. *pneumoniae* infections. Inhibitor experiments revealed that the PI3 kinase activity is important for bacterial entry, while in later stages of infection the anti-apoptotic effect of the enzyme was shown to be important for bacterial survival [44], [26]. The data for *C*. *trachomatis* are somewhat contradictory, as it was shown that entry of serovar L2 EBs is independent of PI3 kinase activity [45]. On the other hand, L2 EBs specifically activate the PI3 kinase by interacting with the host ephrin receptor during internalization [46]. Furthermore, previous studies on Tarp showed that entering L2 EBs colocalize with PI(3,4,5)P produced by an active PI3 kinase, and that Tarp directly interacts with the p85 subunit of the activated kinase [47]. Late in the *C*. *trachomatis* L2 infection a phase of prolonged PI3 kinase signaling, induced by activation of the ephrin receptor, induces an anti-apoptotic state, as has also been shown for *C*. *pneumoniae* [46]. Our data clearly show that activation of the PI3 kinase via EGFR initiates the endocytotic steps required for uptake and survival of *C*. *pneumoniae* EBs.

By employing various marker proteins for endocytotic processes we were able to show by live-cell imaging that the PIP composition of the chlamydial endosomes changes from PI(3,4,5)P to PI3P during the course of internalization. Furthermore, these nascent inclusions also acquire many small Rab GTPases including Rab5, Rab4, Rab11, Rab14 and Rab7 typically found on early or sorting endosomes (SE) (Figs 1–3 and 7, S1 Movie) [6]. These data suggest that the inclusion has become an EE or more likely a SE by 15 min p.i., which is further remodeled to meet chlamydial needs over the following 30 min. That the status of the inclusion as an EE or SE is crucial for successful infection is demonstrated by the negative impact on internalization and infection of inhibition of the PIP phosphatase SHIP2 (Fig 1), which contributes to the conversion of PI(3,4,5)P to PI3P [25].

As the PI3P/Rab5/EEA1 identity of the nascent inclusion remains stable during the first hour p.i., *C*. *pneumoniae* must have evolved a mechanism to ensure that this status is maintained and tightly controlled, as Rab5 recruits Rab7 for vesicle maturation [28]. The fact that at 15 min p.i. the chlamydial vesicle is positive for Rab7, a GTPase regulating lysosomal degradation of proteins, which is subsequently lost supports this idea (Fig 2, S2 Fig) [48]. This finding suggests that, in order to escape lysosomal degradation, *C*. *pneumoniae* either actively releases Rab7 from the endosome or blocks the Rab5/Rab7 switch [28]. Interestingly, in a *C*. *trachomatis* L2 infection of RAW macrophages, Sun and colleagues also showed that EBs associate with both Rab5 and Rab7 by 30 min p.i., and that expression of a dominant negative Rab7 enhanced bacterial replication further underpinning the relevance of Rab7 for infection [49]. Furthermore, when we chemically inhibited PIKfyve, a PIP kinase that phosphorylates the PI3P of EE membranes to PI(3,5)P, which marks late endosomes, we obtained a ~3-fold increase in internalization and infection (Fig 2, S2 Fig) [50], [51]. This inhibitor forces endosomes to remain in the EE state, which is obviously extremely beneficial for internalization and subsequent survival. Moreover, we observed that the EB-containing EEs also lost Rab7 (Figs 2 and 7). Conversely, inhibition of Akt, which is normally activated by the PI3 kinase and itself activates PIKfyve during EGFR endocytosis, leads to an increase in levels of Rab7 on chlamydial endosomes while reducing internalization and infection (Fig 2, S2 Fig) [27]. These results indicate that Rab7 release depends on both blocking PIKfyve-dependent endosome maturation into the LE and on functional Akt activity. Rab7 release results in a chlamydial endosome, which retains early endosomal character and does not fuse with lysosomes, therefore avoiding degradation. How this is achieved remains unclear, but modification of bacteria-containing vesicles and endosome maturation and fate is a common mechanism among intracellular pathogens. Secreted effector proteins are key players in regulating PIP-converting enzymes, or modulating Rab activity by mimicking Rab nucleotide exchange factors (GEFs or GAPs) needed for activity [17], [52].

Intriguingly, besides loss of Rab7, we also observed acquisition of the recycling-related Rab GTPases Rab4, Rab11 and Rab14 immediately after internalization (Figs 3 and 7, S3 Fig). This is in clear contrast to a *C*. *trachomatis* L2 infection, where colocalization with the inclusion was only observed at 2 h p.i. for Rab11 and at 10 h p.i. for Rab14 [18], [20]. Interestingly, while Rab11/Rab14 remained associated with the *C*. *pneumoniae* inclusion membrane from internalization throughout the whole infection cycle, Rab4, like Rab7, is lost from 30 min onwards (Fig 3, S3 Fig). The loss of Rab4 could be due to remodeling of the Chlamydia-containing SE to avoid fast recycling back to the plasma membrane or to establish a specific EE/RE character defined by Rab11/Rab14 [10]. Although Rab4 disappears from the early inclusion, it was found to be again associated with the inclusion membrane of both late *C*. *pneumoniae* and *C*. *trachomatis* infections, and even has a *C*. *trachomatis*-specific interaction partner, the Inc protein CT229 [18], [19]. Together with Rab11 it is thought to be important for the supply of iron to the inclusion [24].

By acquiring Rab11 and Rab14, *C*. *pneumoniae* labels its early inclusion as part of the recycling system of the cell (Fig 3, S3 Fig), since Rab11 is the classical RE-associated Rab protein [11]. How Rab11 is recruited is unclear, but it may require an early effector protein, and indeed the *C*. *pneumoniae*-specific Inc protein Cpn0585 has been shown to interact with Rab11 within the inclusion membrane *late* in infection [23]. And although no specific *C*. *trachomatis* homolog of Cpn0585 is known, Rab11 is a key regulator of Golgi fragmentation and growth of *C*. *trachomatis* [22]. Recruitment of Rabs to vesicular membranes is generally determined by a GTP/GDP switch, regulated by specific GEFs or GAPs, where GTP binding defines the active state that enables membrane association [53], [14]. For internalization and infection by *C*. *pneumoniae*, Rab11 activity is essential, since expression of a dominant-negative Rab11 (S25N), locked in the GDP state, led to fewer internalized EBs and reduced infectivity (Fig 3). Conversely, expression of the constitutively active version (Q70L), which mimics the GTP-bound state, increased internalization and infectivity significantly (Fig 3). Why Rab14, a second specialized Rab protein of the recycling system, is acquired in parallel is still unclear (Figs 3 and 4), but this too seems to be a common theme in pathogenic bacteria [13], [12], [17], [52]. Rab14 has been found to be associated with vacuoles containing *Mycobacterium tuberculosis*, *Legionella pneumophila*, *Coxiella burnetii* and *Klebsiella pneumoniae* [54], [55], [56], [57]. For *C*. *trachomatis* it was shown that Rab14 is recruited to the late inclusion and participates in sphingolipid trafficking from the Golgi [20]. The role of Rab14 in the early *C*. *pneumoniae* infection remains unclear but, by analogy to the *M*. *tuberculosis* infection, it may well act to block maturation of the early inclusion from EE to LE and thereby prevent its early degradation [54].

The data presented here allow to conclude that at 30 min p.i. the internalized *C*. *pneumoniae* EBs can be found in a specialized RE compartment bearing Rab11 and Rab14, with an EE membrane identity. To reach its final destination, the perinuclear region, the chlamydial vesicle must then be transported from the periphery to the center of the cell. As shown here, this is achieved by association of the EB-containing vesicle with the Rab11/Rab14 adaptor protein Fip2 (Figs 4, 5 and 7, S5 Fig) immediately after internalization. Fip2 as a member of the Rab11-FIPs regulates intracellular transport of cargo within the recycling system and was shown to mediate EGFR endocytosis and subsequent endosomal sorting of the receptor [34], [32]. Therefore, Fip2 is essential for a successful infection as (i) it directly influences EGFR-mediated endocytosis and (ii) regulates the intracellular transport of the endocytosed EBs within their specific endosome. This is supported by knockdown of the protein, which significantly impaired internalization and infectivity (Fig 4). Moreover, deletion of Fip2 domains required for binding to membrane lipids (C2), Rab11 (RBD) or the actin motor protein myosin (MyoBD) had a negative influence on internalization of EBs and the intracellular transport of the chlamydial vesicle (Fig 5, S5 Fig). Loss of the C2 domain resulted in vesicular aggregates that were retained in the cell periphery (Fig 5). As the C2 domain preferentially binds to PI(3,4,5)P found in the plasma membrane, the deletion variant cannot interact with the internalized vesicles at the bacterial entry site because its targeting domain is missing [58], [33]. Intracellular transport orchestrated by Rab11 in association with its adaptor proteins is very important in normal cells and in cells infected with intracellular pathogens [11], [59]. Therefore, deletion of the RBD domain had the most drastic effect as bacterial entry was strongly inhibited, and those internalized were retained in the cell periphery and the formed inclusions that remained distant from the nucleus (Fig 5, S5 Fig). Finally, deletion of the myosin Vb-binding domain again resulted in vesicles being retained in the cell periphery and in small underdeveloped inclusions remote from the nucleus (Fig 5, S5 Fig). Moreover, the importance of this actin-specific motor protein for internalization, transport of the early chlamydial vesicle and proper development of *C*. *pneumoniae* was demonstrated by protein knockdown or overexpression of a dominant-negative version (Figs 5 and 6). Both led to reduced internalization and impaired inclusion growth, suggesting that during internalization the chlamydial vesicle associates with Rab11 (Rab14), which interacts with Fip2 which binds to myosin Vb (Fig 7). In this way, the chlamydial vesicle is tethered to the cortical actin cytoskeleton, as endosomes in uninfected cells are captured by myosin Vb, thus retarding their transport to the perinuclear ERC [41], [40]. This tethering is thought to be required for proper transfer of vesicles to microtubules, which then perform the long-distance transport inside the cell [41]. The subsequent microtubule-dependent transport may be regulated by Rab14, as this has an additional adaptor, the kinesin motor protein KIF16b [60], [61].

In conclusion, we show here for the first time how *C*. *pneumoniae* is internalized, progresses through early endocytosis and forms the specialized Chlamydia-containing vacuole by interaction with specific Rab proteins, which together with specific Rab adaptor proteins then establish the intracellular niche.

## Materials and methods

### Inhibitors, antibodies and reagents

Inhibitors against PIKfyve (YM201636) or SHIP2 (AS1949490) were purchased from Calbiochem, LY294002 (PI3 kinase) and MK2206 (Akt kinase) from Selleckchem, AG1478 (Tyrphostin #9842) from Cell Signaling. Primary antibodies against GFP (GF28R), EGFR (#PA1-1110) were purchased from Thermo Scientific, anti MyosinVb (sc-98020) from Santa Cruz, anti Myc (clone 9E10) and anti β-actin (clone AC-15) from Sigma Aldrich and Fip2 (#18136-1-AP) from Proteintech Europe. Directly FITC labeled Pathfinder antibody (#30701) against chlamydial LPS was used from Bio-rad. Antibodies against DnaK were kindly provided by S. Birkelund [62], while antibodies against Pmp21-M or Cpn0147 were generated in our lab [63]. Secondary antibodies for immunofluorescence anti rabbit/ mouse coupled to Alexa 488, 594 or 647 were purchased from Thermo Scientific. Secondary antibodies anti rabbit/ mouse/ goat coupled to alkaline phosphatase for immunoblot detection were purchased from Promega.

### Growth of chlamydia and cell lines

*C*. *pneumoniae* GiD was propagated in HEp-2 cells (ATCC: CCL-23) [64]. HEp-2 and HEK293-FT cells (gift from K. Pfeffer, Medical Microbiology, HHU Düsseldorf) were cultured in DMEM medium supplemented with 10% fetal calf serum (FCS), MEM vitamins and non-essential amino acids (Thermo Scientific). Chlamydial elementary bodies (EBs) were purified using a 30% gastrographin solution (Bayer) and stored in SPG buffer (220 mM sucrose, 3.8 mM KH_2_PO_4_, 10.8 mM Na_2_HPO_4_, 4.9 mM L-glutamine). All cloning was carried out by *in vivo* homologous recombination in *Saccharomyces cerevisiae*. *Escherichia coli* strain XL-1 Blue (Stratagene) was used for plasmid amplification.

### Plasmid constructs, cloning procedures

GFP-Rab5, Rab7 and Rab11 were kindly provided by M. Scidmore [18], GFP-Rab14 and Rab4 by M. Fukuda [65]. Rab11S25N, Rab11Q70L constructs were generated by site-directed mutagenesis using GFP-Rab11 as template. GFP-MyosinVb was kindly provided by J. Roland [66], PH-Btk-GFP by T. Balla [67], and GFP-2xFYVE by H. Stenmark [68]. GFP-EEA1 (#42307) was purchased from Addgene. For lentiviral transduction *CEN-ARS-TRP1* was amplified by PCR and integrated into pWPXL:GFP/mCherry (gift from K. Pfeffer, [69]) to generate yeast shuttle vectors pKM160 and pKM161 respectively. pKM160 served as the backbone for integration of Rab11, Rab11S25N, Rab11Q70L, Fip2, Fip2ΔC2, Fip2ΔRBD, Fip2ΔMyoBD, Fip2-NPF_2_/AAA. pKM161 served as the backbone for integration of 2xFYVE. For transient transfection Rab11-Fip2 and MyoVb were amplified from HEp-2 RNA and integrated into pAE67 (N-terminal GFP). The Fip2 variants Fip2ΔC2, Fip2ΔRBD, Fip2ΔMyoBD, Fip2-NPF_2_/AAA were amplified from wild-type Fip2 and integrated into pAE67. 2xFYVE, Rab11, Fip2 were amplified by PCR and integrated into pAE66 (N terminal mCherry).

### si, miRNA transfection lentiviral transduction

siRNA targeting human Fip2 (#4392420) and control siRNA (#AM4611) were purchased from Ambion. pcDNA6.2GWEmGFP-miR from Thermo Scientific was used to target myosinVb. HEp-2 cells were transiently transfected for 24 to 72 h with siRNA or plasmid DNA using Turbofect (Thermo Scientific). Protein knockdown was analyzed after 72 h by immunoblot analysis of cell extracts following lysis with Phospho-Lysis buffer (1% NP40, 1% Triton X100, 20 mM Tris, 140 mM NaCl, 2 mM EDTA, 1 mM Na_2_VO_4_, Roche Protease Inhibitor Cocktail). Extracts were subjected to SDS/PAGE and target proteins detected with specific primary antibodies and anti-rabbit/mouse or goat coupled to alkaline phosphatase. HEK293 cells were transfected for 48 h with psPAX2, pLVSV-G (gift from K. Pfeffer, [69]) and various pKM160/161 constructs using JetPRIME Polyplus to generate lentiviral particles. HEp-2 cells stably expressing GFP/mCherry were generated by transduction with lentiviral particles and isolated by sorting of cells with FACSAria (BD Bioscience).

### Immunofluorescence staining

Transfected and infected cells were fixed at indicated time points (during early infection, 5 to 60 min p.i.) with 3% paraformaldehyde in PBS (PFA) for 10 min, then washed three times with PBS and permeabilized with either 100% methanol for 10 min or with 2% saponin (Sigma Aldrich) in PBS for 20 min. Depending on the permeabilization protocol, primary antibodies were diluted in PBS or in 0.5% saponin solution and incubated for 30 min at 37°C. Cells were washed three times with PBS with or without 0.5% saponin and incubated with secondary antibodies anti-rabbit/ mouse/goat Alexa488/594/647 for 30 min at 37°C in PBS with or without 0.5% saponin. DAPI was used to visualize DNA.

### Internalization assays of chlamydial particles

Transiently transfected or stably expressing HEp-2 cells were cultivated to 70% confluency in 24-well plates (Sarstedt) on glass coverslips (Ø 1cm^2^), then infected with purified *C*. *pneumoniae* EBs (MOI 5) by centrifugation for 20 min (25°C at 2900 rpm; Rotanda, Hettich). After centrifugation cells were shifted to 37°C and grown under 6% CO_2_ for 5 to 60 min, washed three times with PBS and fixed with 3% paraformaldehyde in PBS (PFA) for 10 min and permeabilized to analyze endosome or Rab distribution by confocal microscopy (Nikon Confocal C2plus). Internalization rates were determined either by immunostaining or by q-PCR of cells infected for 2 h. Briefly, for microscopic analyses cells were fixed with PFA for 10 min, washed three times with PBS and stained with anti-Pmp21-M and anti-rabbit Alexa488 or 594 and DAPI. Cells were imaged by confocal microscopy and internalization ratios were determined by counting external Pmp21-positive and all DAPI-positive bacteria. For q-PCR-based analyses cells were trypsinized after 2 h of infection, and pelleted for 10 min at 500 x g. Total DNA was isolated by phenol extraction (Roth), and internalization of EBs was determined by q-PCR using primers for human GAPDH and chlamydial 16S RNA.

### Infection experiments

HEp-2 pretreated with inhibitor or transfected with siRNAs, miRNAs or GFP/mCherry constructs were infected with *C*. *pneumoniae* EBs (MOI 1) by short centrifugation as described before, then shifted to 37°C for 2 h before the infection medium was replaced by fresh medium (transfected cells) or fresh medium supplemented with 1.2 μg/ml cycloheximide, and incubated for 48 h. The number of inclusions formed was quantified by confocal imaging, using either an FITC-conjugated antibody directed against chlamydial LPS (Pathfinder) or antibodies directed against Cpn0147, as described previously.

### Co-immunoprecipitation of endosomes from infected cells

Transiently transfected or stably expressing HEp-2 cells were cultivated to 90% confluency in 6-well plates (Sarstedt), then infected with purified *C*. *pneumoniae* EBs (MOI 100) by centrifugation as described previously. After centrifugation cells were shifted to 37°C and incubated under 6% CO_2_ for 15 min, washed three times with PBS and detached in PBS using a cell scraper (Sarstedt). Cells were lysed by passing them through a G-21 needle (Braun) 15 times, and centrifuged for 10 min at 4°C at 500 x g to obtain a post-nuclear supernatant (PNS) containing intact endosomal vesicles. The supernatant was mixed with 50 μl Protein G Dynabeads (Thermo Scientific) previously incubated with 5 μg anti-GFP or anti-Myc antibody O/N at 4°C. Co-IP was performed according to the manufacturer’s protocol, and eluted proteins were resolved by SDS/PAGE and detected by immunoblot.

### Microscopy of live cells

Transiently transfected or stably expressing HEp-2 cells were grown to 70% confluency in μ-Dish 35 mm glass bottom chambers (ibidi). Purified *C*. *pneumoniae* EBs (MOI 50) were incubated for 10 min in PBS containing 0.5 μg/ml Hoechst 33342 (Thermo Scientific), then washed once with PBS. Cells were washed three times with imaging buffer (120 mM NaCl_2_, 25 mM HEPES, 3 mM KCl_2_, 2 mM CaCl_2_, 3 mM NaHCO_3_, 2 mM MgCl_2_, 2 mM pyruvic acid, 5 mM glucose) and infected with Hoechst-stained *C*. *pneumoniae* EBs in imaging buffer by short centrifugation. Chambers were transferred to Nikon Confocal C2plus and continuously imaged with 3 to 5 z-planes using Perfect Focus System for 30 to 60 min at 37°C.

### Microscopy and image processing

All imaging was performed using an inverse Nikon TiE Live Cell Confocal C2plus with 100 x TIRF objective and a C2 SH C2 Scanner. For life cell imaging a heat controled incubator (Pecon) and heated objective were used. All images, movies and image related measurements were generated with Nikon NIS Elements software.

### Statistical analysis

The data represent the mean±SD of *n* experiments. For simple paired analyses between two groups, a Student's *t*-test was chosen. A *P* value of less than 0.01 was considered to be statistically significant.

### Accession numbers

*Homo sapiens* Epidermal growth factor receptor (EGFR): NM_005228.3
*Homo sapiens* Rab5: M28215.1
*Homo sapiens* Rab4a: AF498934.1
*Canis lupus* Rab7a: NM_001003316.1
*Homo sapiens* Rab11a: AF000231.1
*Homo sapiens* Rab14: NM_016322.3
*Homo sapiens* Rab11-Fip2: NM_014904.2
*Homo sapiens* EEA1: NM_003566.3
Homo sapiens MyosinVb: NM_001080467.2
*Chlamydia pneumoniae* GiD: LN847009.1

## Supporting information

S1 Fig Entry of *C*. *pneumoniae* EBs is PI3K dependent. **(A)** Quantification of infection in cells preincubated with LY29 (50 μmol) or equal amounts of DMSO for 2 h prior to infection. Cells were infected with a MOI 1, fixed at 48hpi and stained with FITC-labeled LPS antibodies and DAPI. Inclusions were counted in 40 visual fields (n = 4). **(B)** Quantification of colocalization of EGFR-positive EBs with GFP-Rab5 or GFP-EEA1 during the first hour of infection as described previously. Confocal images of 30 individual cells were analyzed (n = 3). **(C)** Colocalization of EGFR stained with anti EGFR and anti-rabbit Alexa594, *C*. *pneumoniae* EBs stained with DAPI at 15 min p.i. in cells expressing GFP-2xFYVE (top row), GFP-EEA1 (middle row) or GFP-Rab5 (bottom row). White arrows indicate EBs colocalizing with EGFR and EE markers tagged with GFP. Bar 1μm. **(D)** Quantification of infection in cells preincubated with SHIP2 inhibitor (10 μmol) equal amounts DMSO at 48 hpi as described above (n = 4). *** *P* value ≤0.001.
(TIF)

S2 Fig Development of the early *C*. *pneumoniae* inclusion is dependent on Akt/PIKfyve activity. **(A, B)** Quantification of infection in cells preincubated with Akt- or PIKfyve-specific inhibitors or equal amounts of DMSO for 2 h prior to infection. Cells were infected at MOI 1, fixed at 48 hpi and stained with FITC-labeled LPS antibodies and DAPI. Inclusions were counted in 40 visual fields. **(A)** Degree of inhibition of infection by pre-incubation with the Akt inhibitor MK22 (3 μmol) (n = 4). **(B)** Quantification of infection in cells pretreated with the PIKfyve inhibitor (800 nmol) (n = 4). **(C)** Confocal images of colocalization of GFP-Rab7 with EBs (DAPI) in PI3P-positive endosomes (visualized with mCherry-2xFYVE) at 30 min p.i. in cells treated with DMSO (top row), MK22 (middle row) or the PIKfyve inhibitor (bottom row) prior to infection. White arrows indicate colocalization. Bar 1 μm. *** *P* value ≤0.001.
(TIF)

S3 Fig The early *C*. *pneumoniae* inclusion is a recycling endosome. **(A-C)** Confocal images of GFP-tagged Rab11, Rab4 and Rab14 with *C*. *pneumoniae* EBs stained by DAPI and endogenous EGFR stained by anti EGFR and anti-rabbit Alexa594 at 15 min (top row images) and 30 min p.i. (bottom row images). White arrow indicate colocalization. Bar 1μm. **(A)** Colocalization of Rab11 and EGFR. **(B)** Colocalization of Rab4 and EGFR. **(C)** Colocalization of Rab14 and EGFR.
(TIF)

S4 Fig The Rab11/Rab14 adaptor Fip2 is recruited to early *C*. *pneumoniae* inclusions. **(A)** Confocal images of *C*. *pneumoniae* EBs stained with DAPI colocalizing with GFP-Fip2 and mCherry-Rab11 (top row) or with GFP-Rab14 and mCherry-Rab11 (bottom row) at 15 min p.i. White arrows indicate colocalization. Bar 1 μm. **(B, C)** Immunoblot analyses of Co-IP s obtained from cells transfected with EGFR-Myc and GFP-Fip2 **(B)** or GFP-Rab11 **(C)** infected for 15 min with *C*. *pneumoniae* EBs for 15 min or incubated with a low (1 ng/ml; +) or a high (100 ng/ml; ++) concentration of EGF. Equal amounts of sample taken from the Input and Elution fractions were loaded. **(B)** Endosomes of EGFR-Myc- and GFP-Fip2-expressing cells were isolated after 15 min and immunoprecipitated with an anti-Myc antibody and analyzed by immunoblot using anti-Myc, anti-GFP and anti-DnaK antibodies. Cell lysate from cells infected for 72 h served as control (last lane). Arrows mark specific protein bands, the asterisk indicates unspecific bands detected in the infected cells by the DnaK antibody. **(C)** Immunoblot analysis of Co-IP obtained from EGFR-Myc- and GFP-Rab11-expressing cells. **(D)** Confocal images of colocalization of GFP-Fip2, mCherry-Rab11 and the *C*. *pneumoniae* inclusion membrane stained with anti-Cpn0147 and anti-rabbit Alexa647 at 48 hpi. Bacterial DNA was visualized with DAPI. Bar 10 μm.
(TIF)

S5 Fig The Rab11 binding domain of Fip2 is essential for the *C*. *pneumoniae* infection. **(A, B)** Quantification of the relative inclusion diameter **(A)** or mean distance of inclusion to nucleus **(B)** in HEp-2 cells stably expressing GFP-Fip2 mutant variants at 30 h p.i. On average, 50 inclusions were measured using confocal images and the Nikon NHI Elements software tool. (n = 3) **(C)** Confocal images of GFP-Fip2-, GFP-Fip2ΔC2-, GFP-Fip2ΔRBD- and GFP-Fip2ΔMyoBD-expressing cells used in **(A, B)** at 30 h p.i. The inclusion membrane was stained with anti Cpn0147 and anti-rabbit Alexa594. DNA was visualized with DAPI. White arrows indicate inclusion localization. Bar 10 μm. *** *P* value ≤0.001, n.s. *P* value ≤0.01.
(TIF)

S1 Movie Live imaging of cells transfected with Btk-PH-GFP and mCherry-2xFYVE that had been infected with living Hoechst-labeled *C*. *pneumoniae* EBs by a short centrifugation. Cells were immediately transferred to a temperature-controlled chamber and imaged continuously with 3-z stacks. The movie shows colocalization of PI(3,4,5)P, PI3P and chlamydial DNA in one z-stack. Original duration of the clip is 5 min and it starts at 0 min p.i.
(AVI)

S2 Movie Live imaging of cells transfected with EGFR-GFP and mCherry-2xFYVE that had been infected with living Hoechst-labeled *C*. *pneumoniae* EBs by a short centrifugation. Cells were transferred to a temperature-controlled chamber and imaged continuously with 3-z stacks. The movie shows colocalization of EGFR, PI3P and chlamydial DNA in one z-stack. Original duration of the clip is 10 min and it starts at 0 min p.i.
(AVI)

S3 Movie Live imaging of cells transfected with EGFR-GFP and mCherry-2xFYVE that had been infected with living Hoechst-labeled *C*. *pneumoniae* EBs by a short centrifugation. Cells were transferred to a temperature-controlled chamber and imaged continuously with 3-z stacks. The movie shows colocalization of EGFR, PI3P and chlamydial DNA in one z-stack. Original duration of the clip is 10 min and it starts at 0 min p.i.
(AVI)

S4 Movie Live imaging of cells transfected with GFP-Fip2 and EGFR-mCherry, and infected with *C*. *pneumoniae* EBs labeled with Hoechst. Cells were imaged continuously with 5-z stacks. The movie shows colocalization of EGFR, Fip2 and chlamydial DNA in one z-stack. Original time is 10 min.
(AVI)

S5 Movie Live imaging of cells transfected with GFP-Fip2 and mCherry-2xFYVE were infected as described in the legend to S3 Fig. Cells were imaged continuously with 3-z stacks. The movie shows colocalization of Fip2, PI3P and chlamydial DNA in one z-stack. Original time is 10 min.
(AVI)

S6 Movie Live imaging of cells transfected with GFP-Fip2 and mCherry-2xFYVE were infected as described in the legend to S3 Fig. Cells were imaged continuously with 3-z stacks. The movie shows colocalization of Fip2, PI3P and chlamydial DNA in one z-stack. Original time is 10 min.
(AVI)

S7 Movie Live imaging of cells transfected with GFP-MyoVbtail and mCherry-2xFYVE and infected with *C*. *pneumoniae* EBs as described in the legend to S3 Fig. Cells were imaged continuously with 3-z stacks. The movie shows colocalization of MyoVbtail, PI3P and chlamydial DNA in one z-stack. Original time is 10 min.
(AVI)
