# Supplementary figures and images for: Acquisition of Rab11 and Rab11-Fip2—A novel strategy for Chlamydia pneumoniae early survival
Source: PLoS Pathog. 2017 Aug 7;13(8):e1006556. doi: 10.1371/journal.ppat.1006556 (PMC5560749; doi:10.1371/journal.ppat.1006556)

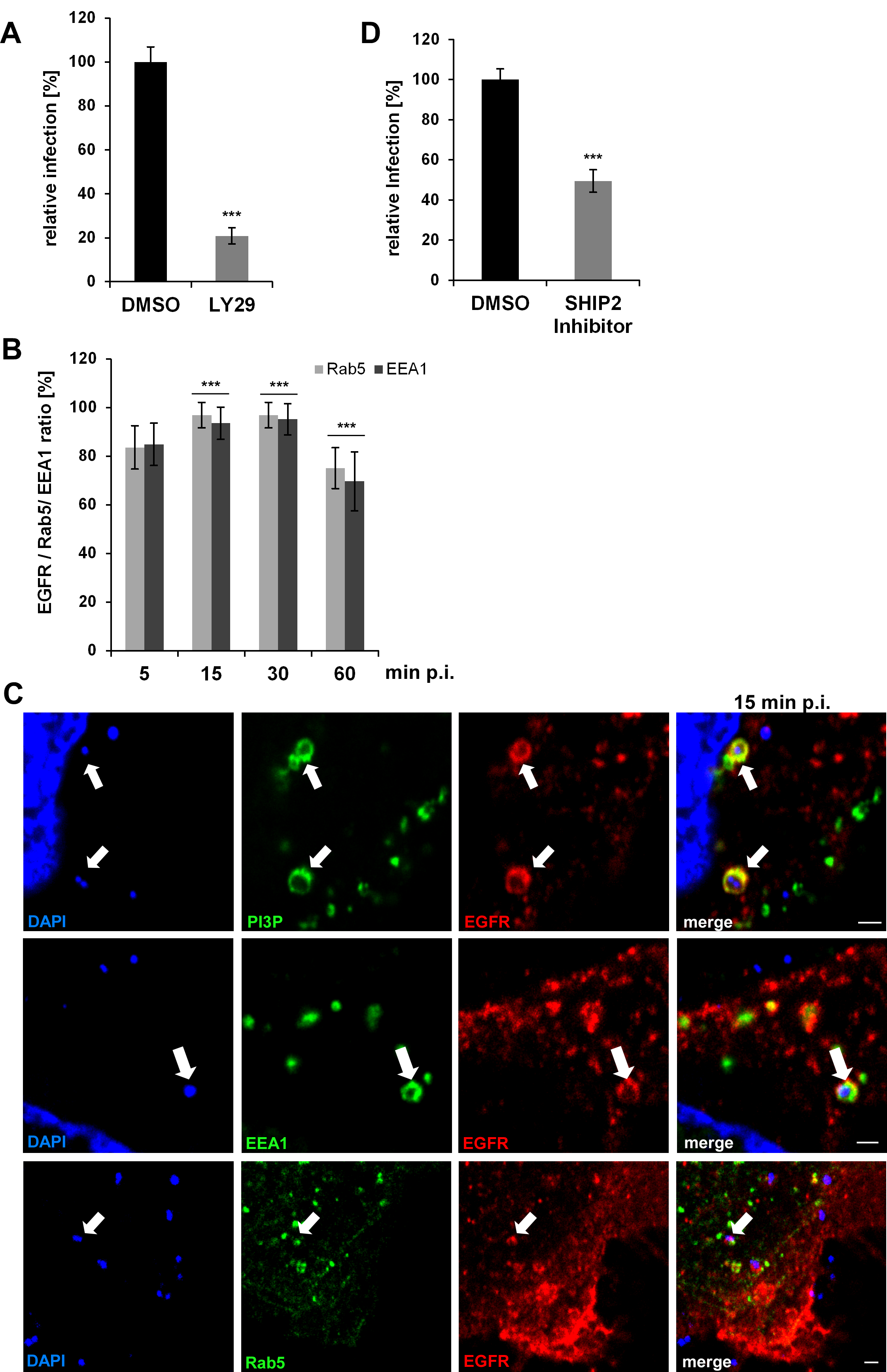

Supplement: S1 Fig — (A) Quantification of infection in cells preincubated with LY29 (50 μmol) or equal amounts of DMSO for 2 h prior to infection. Cells were infected with a MOI 1, fixed at 48hpi and stained with FITC-labeled LPS antibodies and DAPI. Inclusions were counted in 40 visual fields (n = 4). (B) Quantification of colocalization of EGFR-positive EBs with GFP-Rab5 or GFP-EEA1 during the first hour of infection as described previously. Confocal images of 30 individual cells were analyzed (n = 3). (C) Colocalization of EGFR stained with anti EGFR and anti-rabbit Alexa594, C. pneumoniae EBs stained with DAPI at 15 min p.i. in cells expressing GFP-2xFYVE (top row), GFP-EEA1 (middle row) or GFP-Rab5 (bottom row). White arrows indicate EBs colocalizing with EGFR and EE markers tagged with GFP. Bar 1μm. (D) Quantification of infection in cells preincubated with SHIP2 inhibitor (10 μmol) equal amounts DMSO at 48 hpi as described above (n = 4). *** P value ≤0.001. (TIF) [file ppat.1006556.s001.tif]

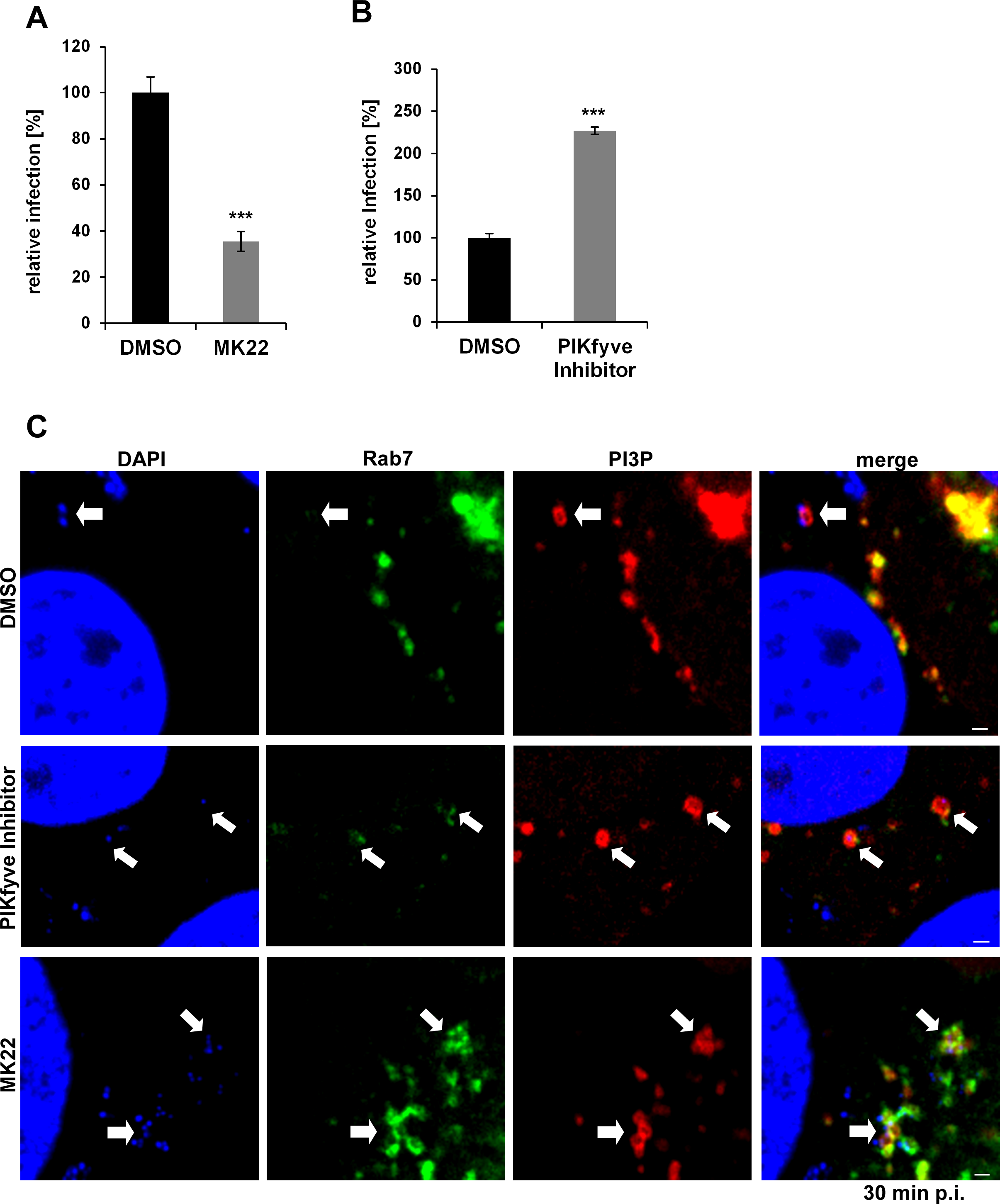

Supplement: S2 Fig — (A, B) Quantification of infection in cells preincubated with Akt- or PIKfyve-specific inhibitors or equal amounts of DMSO for 2 h prior to infection. Cells were infected at MOI 1, fixed at 48 hpi and stained with FITC-labeled LPS antibodies and DAPI. Inclusions were counted in 40 visual fields. (A) Degree of inhibition of infection by pre-incubation with the Akt inhibitor MK22 (3 μmol) (n = 4). (B) Quantification of infection in cells pretreated with the PIKfyve inhibitor (800 nmol) (n = 4). (C) Confocal images of colocalization of GFP-Rab7 with EBs (DAPI) in PI3P-positive endosomes (visualized with mCherry-2xFYVE) at 30 min p.i. in cells treated with DMSO (top row), MK22 (middle row) or the PIKfyve inhibitor (bottom row) prior to infection. White arrows indicate colocalization. Bar 1 μm. *** P value ≤0.001. (TIF) [file ppat.1006556.s002.tif]

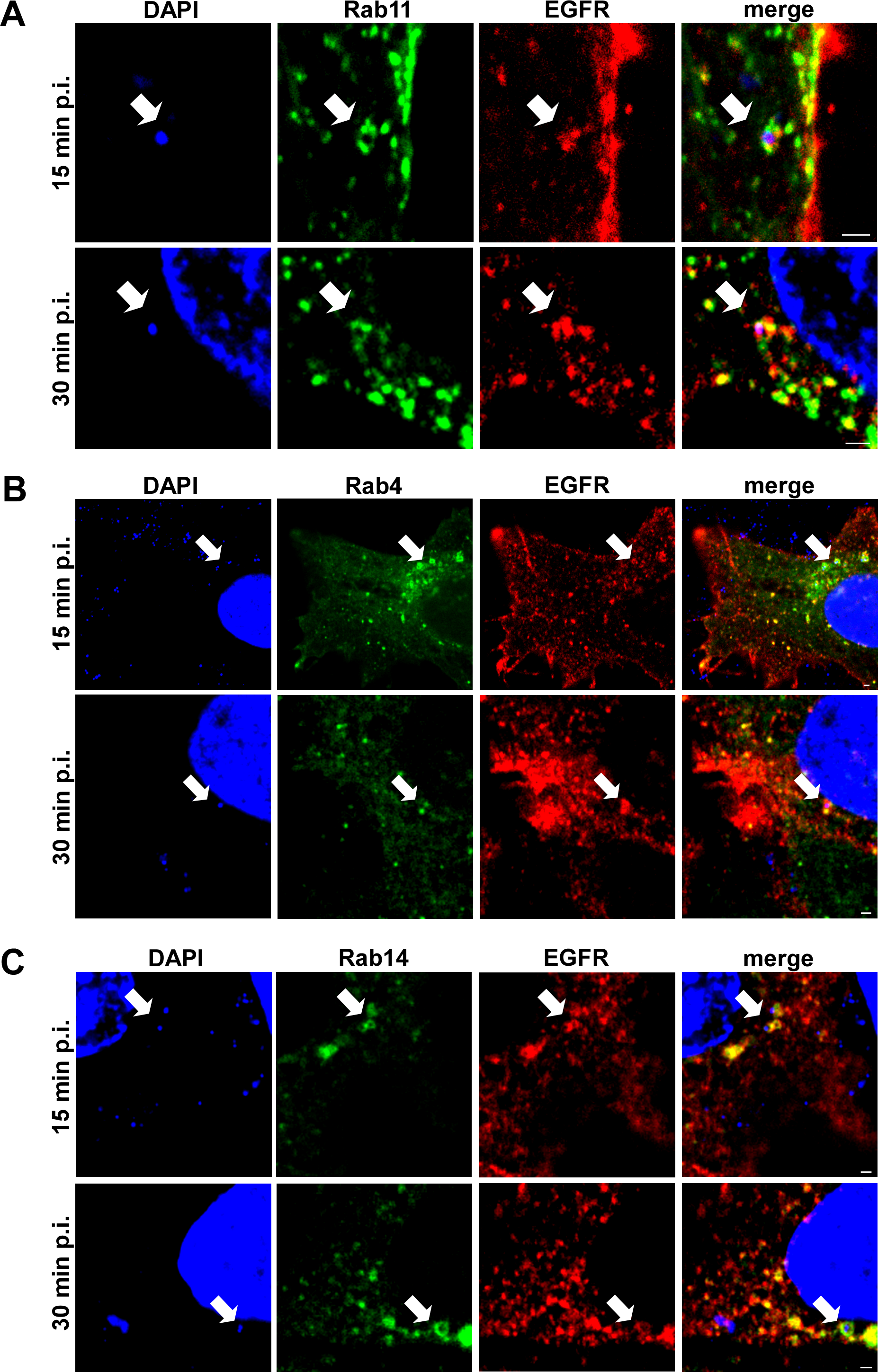

Supplement: S3 Fig — (A-C) Confocal images of GFP-tagged Rab11, Rab4 and Rab14 with C. pneumoniae EBs stained by DAPI and endogenous EGFR stained by anti EGFR and anti-rabbit Alexa594 at 15 min (top row images) and 30 min p.i. (bottom row images). White arrow indicate colocalization. Bar 1μm. (A) Colocalization of Rab11 and EGFR. (B) Colocalization of Rab4 and EGFR. (C) Colocalization of Rab14 and EGFR. (TIF) [file ppat.1006556.s003.tif]

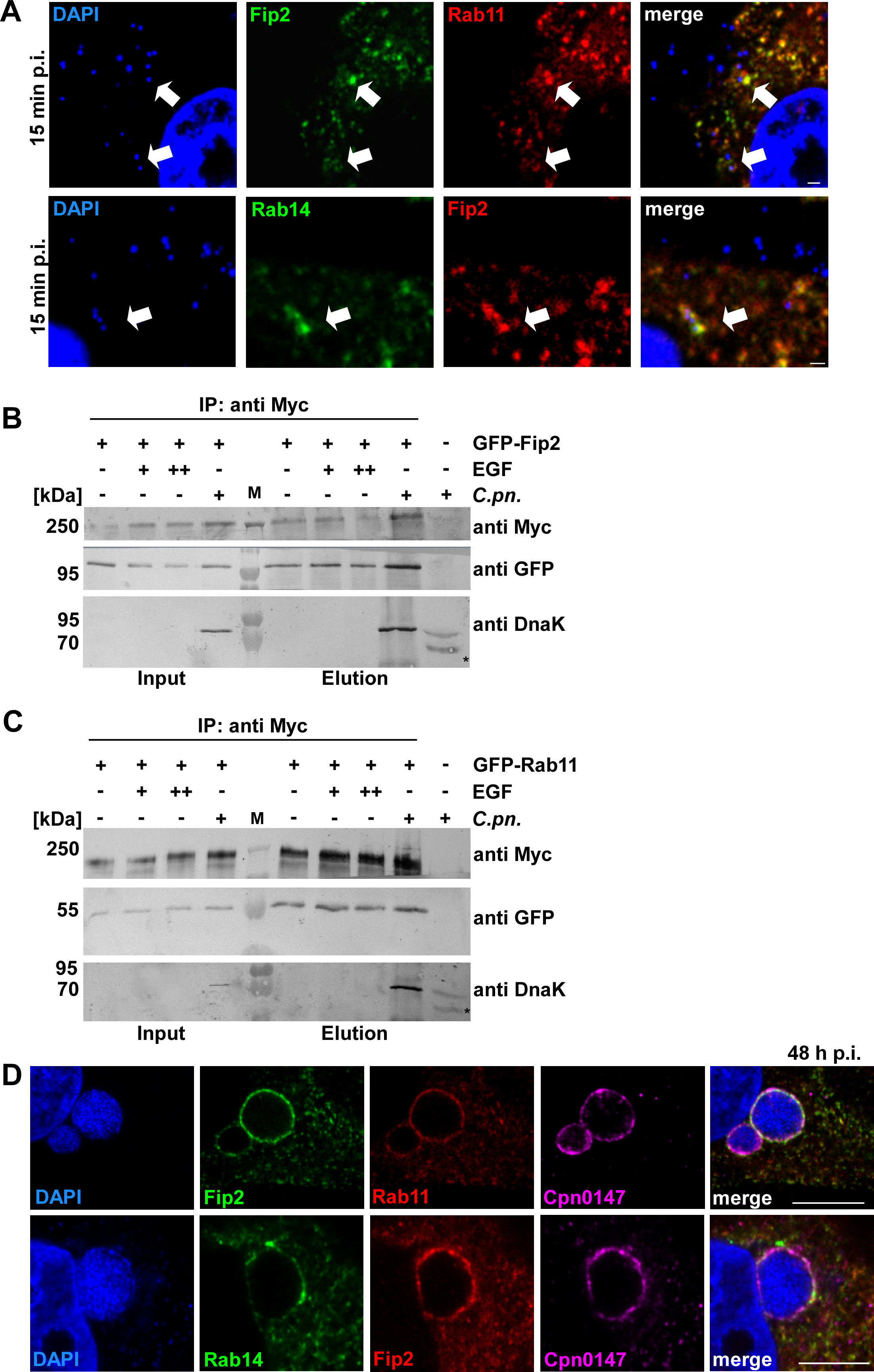

Supplement: S4 Fig — (A) Confocal images of C. pneumoniae EBs stained with DAPI colocalizing with GFP-Fip2 and mCherry-Rab11 (top row) or with GFP-Rab14 and mCherry-Rab11 (bottom row) at 15 min p.i. White arrows indicate colocalization. Bar 1 μm. (B, C) Immunoblot analyses of Co-IP s obtained from cells transfected with EGFR-Myc and GFP-Fip2 (B) or GFP-Rab11 (C) infected for 15 min with C. pneumoniae EBs for 15 min or incubated with a low (1 ng/ml; +) or a high (100 ng/ml; ++) concentration of EGF. Equal amounts of sample taken from the Input and Elution fractions were loaded. (B) Endosomes of EGFR-Myc- and GFP-Fip2-expressing cells were isolated after 15 min and immunoprecipitated with an anti-Myc antibody and analyzed by immunoblot using anti-Myc, anti-GFP and anti-DnaK antibodies. Cell lysate from cells infected for 72 h served as control (last lane). Arrows mark specific protein bands, the asterisk indicates unspecific bands detected in the infected cells by the DnaK antibody. (C) Immunoblot analysis of Co-IP obtained from EGFR-Myc- and GFP-Rab11-expressing cells. (D) Confocal images of colocalization of GFP-Fip2, mCherry-Rab11 and the C. pneumoniae inclusion membrane stained with anti-Cpn0147 and anti-rabbit Alexa647 at 48 hpi. Bacterial DNA was visualized with DAPI. Bar 10 μm. (TIF) [file ppat.1006556.s004.tif]

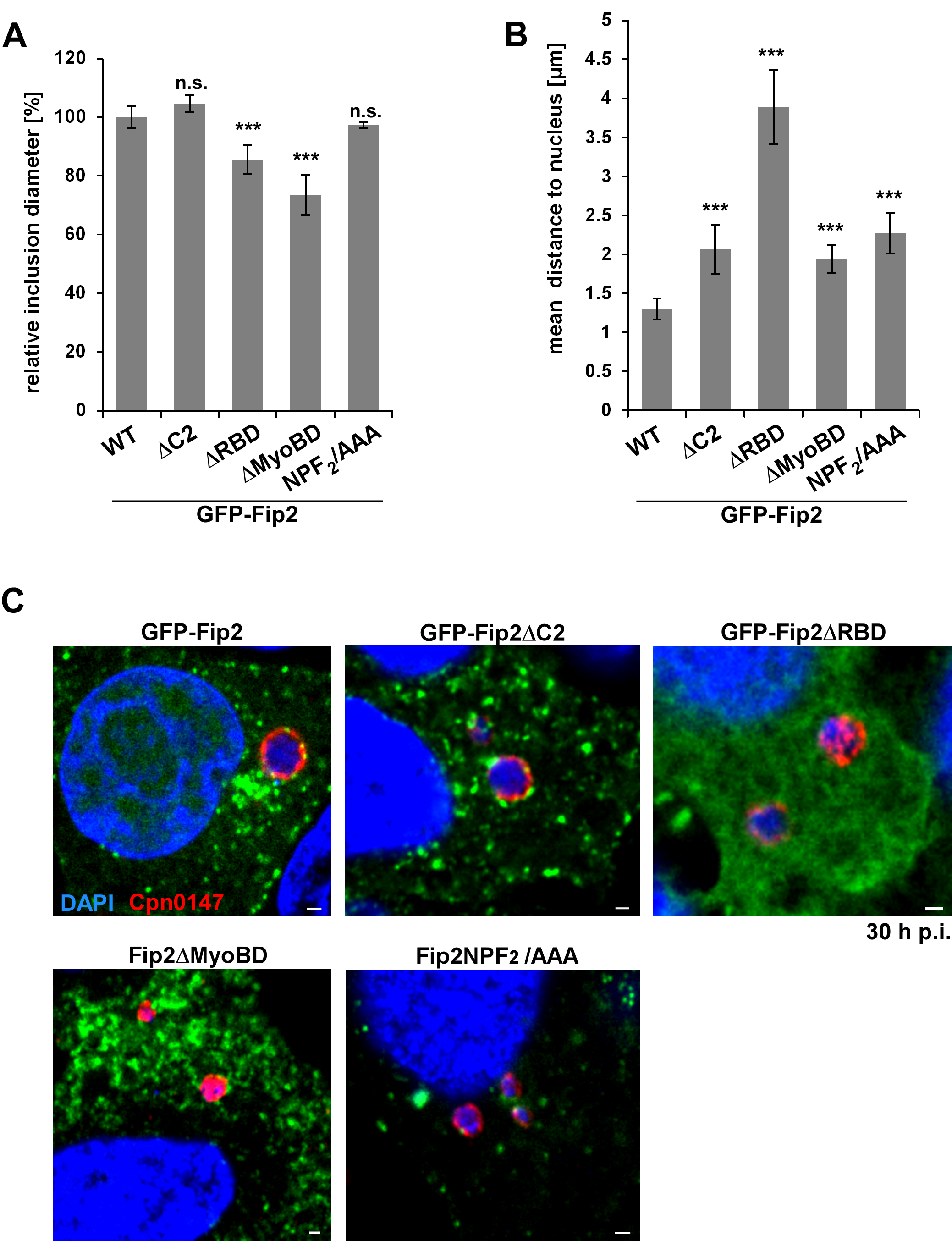

Supplement: S5 Fig — (A, B) Quantification of the relative inclusion diameter (A) or mean distance of inclusion to nucleus (B) in HEp-2 cells stably expressing GFP-Fip2 mutant variants at 30 h p.i. On average, 50 inclusions were measured using confocal images and the Nikon NHI Elements software tool. (n = 3) (C) Confocal images of GFP-Fip2-, GFP-Fip2ΔC2-, GFP-Fip2ΔRBD- and GFP-Fip2ΔMyoBD-expressing cells used in (A, B) at 30 h p.i. The inclusion membrane was stained with anti Cpn0147 and anti-rabbit Alexa594. DNA was visualized with DAPI. White arrows indicate inclusion localization. Bar 10 μm. *** P value ≤0.001, n.s. P value ≤0.01. (TIF) [file ppat.1006556.s005.tif]
